# Neuromelanin organelles are specialized autolysosomes that accumulate undegraded proteins and lipids in aging human brain and are likely involved in Parkinson’s disease

**DOI:** 10.1038/s41531-018-0050-8

**Published:** 2018-06-05

**Authors:** Fabio A. Zucca, Renzo Vanna, Francesca A. Cupaioli, Chiara Bellei, Antonella De Palma, Dario Di Silvestre, Pierluigi Mauri, Sara Grassi, Alessandro Prinetti, Luigi Casella, David Sulzer, Luigi Zecca

**Affiliations:** 1 Institute of Biomedical Technologies, National Research Council of Italy, Segrate, Milan, Italy; 2 IRCCS Don Carlo Gnocchi ONLUS Foundation, Milan, Italy; 3 Department of Medical Biotechnology and Translational Medicine, University of Milan, Segrate, Milan, Italy; 4 Department of Chemistry, University of Pavia, Pavia, Italy; 5 Department of Psychiatry, Columbia University Medical Center, New York State Psychiatric Institute, New York, NY USA; 6 Department of Neurology, Columbia University Medical Center, New York, NY USA; 7 Department of Pharmacology, Columbia University Medical Center, New York, NY USA

## Abstract

During aging, neuronal organelles filled with neuromelanin (a dark-brown pigment) and lipid bodies accumulate in the brain, particularly in the substantia nigra, a region targeted in Parkinson’s disease. We have investigated protein and lipid systems involved in the formation of these organelles and in the synthesis of the neuromelanin of human substantia nigra. Membrane and matrix proteins characteristic of lysosomes were found in neuromelanin-containing organelles at a lower number than in typical lysosomes, indicating a reduced enzymatic activity and likely impaired capacity for lysosomal and autophagosomal fusion. The presence of proteins involved in lipid transport may explain the accumulation of lipid bodies in the organelle and the lipid component in neuromelanin structure. The major lipids observed in lipid bodies of the organelle are dolichols with lower amounts of other lipids. Proteins of aggregation and degradation pathways were present, suggesting a role for accumulation by this organelle when the ubiquitin-proteasome system is inadequate. The presence of proteins associated with aging and storage diseases may reflect impaired autophagic degradation or impaired function of lysosomal enzymes. The identification of typical autophagy proteins and double membranes demonstrates the organelle’s autophagic nature and indicates that it has engulfed neuromelanin precursors from the cytosol. Based on these data, it appears that the neuromelanin-containing organelle has a very slow turnover during the life of a neuron and represents an intracellular compartment of final destination for numerous molecules not degraded by other systems.

## Biochemistry: Mummified organelles burying cellular debris

Subcellular compartments in the brain that contain a dark brown pigment, known as neuromelanin, function like a warehouse for a broad array of molecules not degraded by other cellular cleanup systems. Luigi Zecca from the National Research Council of Italy and colleagues characterized all of the proteins and lipid molecules found inside neuromelanin-containing organelles of the substantia nigra, a region of the human brain that is lost in Parkinson’s disease. They identified many proteins involved in the breakdown of molecules — a sign that this organelle is a degradative organelle known as an autolysosome. Interestingly, the number of these proteins was lower than in a typical lysosome, suggestive of impaired degradative ability. The authors conclude that these neuromelanin-containing organelles accumulate pigment, lipids, proteins, and other compounds over the lifetime of a neuron, possibly leading to cellular dysfunction and disease.

## Introduction

**Fig. 1:**
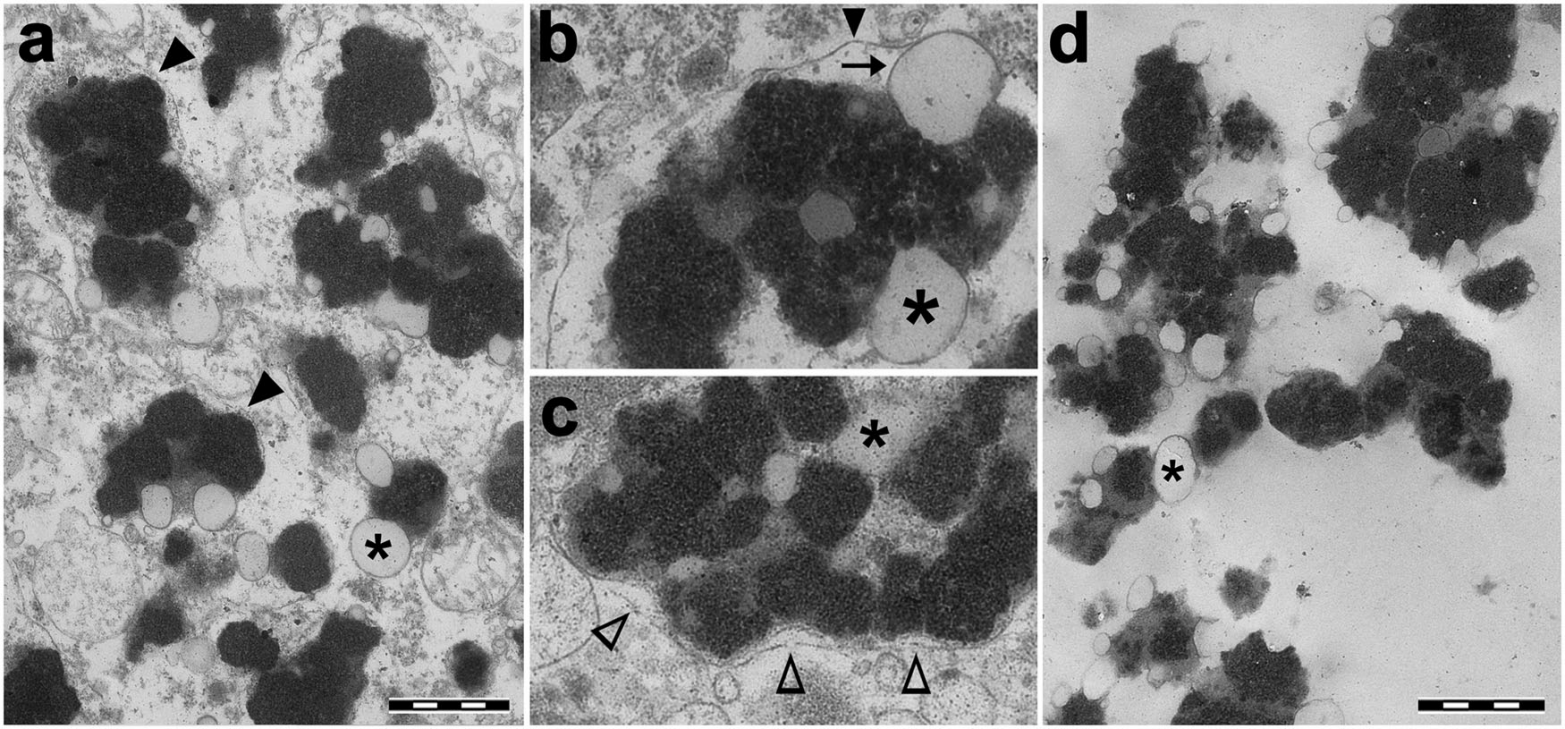
Transmission electron microscopy images of NM-containing organelles in human SN tissue (**a**–**c**) and after the isolation procedure (**d**). **a**–**c** SN tissue of 89 y.o. healthy subject. Intraneuronal NM-containing organelles of the SN are membrane bounded (black arrowhead in **a** and **b**) and contain large amounts of NM pigment (black and electron dense), a protein matrix and lipid bodies (asterisk). Scale bar =1 µm in **a**. Large lipid bodies (asterisk) are surrounded by a membrane as demonstrated in **b** (arrow), although the images do not distinguish between a bilayer and single layer membrane. Considering that brain samples used in this study were *post mortem* tissues, it is striking that there is often a double membrane around many of the organelles. At higher magnification, a double membrane delimiting NM-containing organelle is clearly visible (empty arrowhead in **c**). **d** NM-containing organelles isolated from the SN tissue of 89 y.o. healthy subject (the same subject of **a**–**c**). The purity and integrity of isolated NM-containing organelles is clearly demonstrated by transmission electron microscopy: low magnification **d** demonstrates that cellular and subcellular debris are completely absent. The outer limiting membrane is not apparent, but the constituents of the organelles appear intact with NM pigment, many lipid bodies (asterisks) and membranes of lipid bodies. Scale bar = 1 µm in **d**

Electron microscopy studies of neurons of numerous brain regions have demonstrated that organelles containing neuromelanin (NM) exhibit abundant clear “lipid bodies” (sometimes referred to as “lipid droplets” in the literature, although this term is widely used for a different lipid storage organelle) and a dark electron-dense matrix.^1–3^ The number of these organelles and the concentration of NM pigment increase linearly during aging.^3–6^ These organelles are highly concentrated in dopamine (DA) neurons of the substantia nigra (SN) (Fig. 1a,b,c) and norepinephrine neurons of locus coeruleus,^2,5,6^ brain regions strongly targeted in Parkinson’s disease (PD).^7,8^ The pigments of these organelles are a family of compounds formed by a melanic, aliphatic, and protein components with variable ratios.^9^ NM pigment also accumulates large amounts of metals, further confirming that these organelles continuously accumulate in aging due to very slow turnover.^3^

The formation of NM appears to provide a protective process,^3,10^ but the amount of NM accumulated in neurons is related to their vulnerability in PD.^8,11,12^ Due to its biochemical properties, NM has long been suggested as a critical factor underlying neuronal vulnerability in PD.^8^ Indeed, NM is suggested to play a dual role, both toxic and protective, that is determined by the cellular context and conditions.^13,14^ The synthesis of NM is neuroprotective since it removes from the cytosol the reactive/toxic quinones that would otherwise induce neurotoxicity.^10^ NM further plays a protective role by chelating potentially toxic metals, including Fe, Zn, Cu, Al, Cr, Mo, Pb, and Hg (refs. ^3,15^), drugs and organic toxicants.^16–18^ However, NM can play a toxic role when released by degenerating neurons of the SN during PD: under these conditions, NM acutely discharges high amounts of metals and organic chemicals accumulated over many years of life. NM released by degenerating neurons in PD activates microglia, producing reactive and pro-inflammatory molecules that induce further neuronal death and release of NM, thus establishing a vicious cycle of neuroinflammation and neurodegeneration.^19^ The activation of microglia by NM can drive antigen presentation by SN and locus coeruleus catecholaminergic neurons, a response that may play a crucial role in PD pathogenesis.^20^ NM can also stimulate dendritic cells in vitro inducing their maturation.^21^

The structure of the melanic component is different in various types of NM pigments,^9,22^ and multiple features of NM structure, as well as protein and lipid composition of NM-containing organelles, remain incompletely characterized. Indeed, the protein components of NM-containing organelle have been partially characterized, and these are consistent with its lysosomal nature.^23,24^ However, the characterization of proteins belonging to different portions of the NM-containing organelle and their localization are yet to be clarified, although this knowledge is fundamental to understanding the complex nature of these organelles. Current data do not indicate the mechanisms of NM accumulation, protein and lipid transport and accumulation within the organelle, or the role of these organelles inside neurons. It is further unknown whether the NM pigment is synthesized within these organelles or transported inside after synthesis elsewhere.

Here we investigate the proteins and lipids of NM-containing organelles from human SN and their accumulation inside these organelles. The NM-containing organelle analyzed here is a paradigmatic case in which an abundant accumulation of the melanic pigment occurs; however, the presence of these organelles is observed throughout the entire brain as a result of physiological aging.^3^

The aim of this study is to perform an extended characterization of proteins and lipids of NM-containing organelles from human SN. To this end, we required highly purified preparations of NM-containing organelles. In order to control contaminations and to avoid the loss of some proteins or lipids, we prepared three types of NM-containing samples with different procedures and compared the data obtained by liquid chromatography-mass spectrometry (LC-MS) determinations on these samples. To further confirm the reliability of our results, independent determinations were also made by immunoelectron microscopy (IEM), western blotting (WB), and thin-layer chromatography (TLC) in addition to LC-MS. In this study, protein profiles were analyzed (i) in three preparations derived from human SN, the organelles containing NM pigment (ORG), NM pigment purified from SN tissues (TIS-NM), and NM pigment isolated from organelles (ORG-NM) using LC-MS; (ii) by WB of ORG samples; and (iii) by IEM of SN tissue slices. The lipid pathways were analyzed by TLC and LC-MS analyses of lipid molecules associated with lipid bodies and with NM pigment, and through characterization of transport proteins and related enzymes by LC-MS, IEM, and WB analyses.

The combination of proteomics, lipidomics, imaging, and biochemical techniques in the study of NM-containing organelles is required for a detailed description of the molecular mechanisms associated with brain aging and neurodegeneration. Previous studies have only partially addressed these issues.^23–27^ The identification of these pathways is crucial for elucidating the processes mediating neuronal survival and vulnerability during aging and PD.

## Results

### NM organelles proteins were identified by analyzing three types of samples: isolated NM-containing organelles, NM purified from SN tissues, and NM purified from NM-containing organelles

The purpose of this study is to describe the proteins present inside the NM-containing organelles, and then distinguish those covalently bound to NM pigment from those not attached to NM. The proteins bound to NM pigment may be more related to the initial steps of NM synthesis, while those not bound to NM pigment may play a role in the membrane, transport, and storage processes involved in NM-containing organelle formation. There are several experimental limitations in the analyses of proteins in NM-containing organelles. In human *post mortem* brain, the membranes of organelles undergo degradation, and their constituents can be released from organelles to the cytosol, and conversely, the organelles can be contaminated by cytosolic components. Furthermore, when processing brain tissues for isolation of ORG samples, the membranes can be broken with consequent mixing of intra- and extracellular proteins. The washing procedure of ORG isolation can moreover change the protein content of organelles due to membrane damage. The native proteins detected by analyzing ORG samples therefore likely underestimate the original protein content, while there may be an increased number of proteins due to contamination.

Thus, we also isolated TIS-NM for protein characterization by LC-MS. During the isolation process of TIS-NM, membranes are broken and additional proteins are likely aspecifically adsorbed by NM pigment. TIS-NM was prepared following the procedure reported by several previous studies.^3,9,28^ Here, proteinase K was employed during the isolation procedure of TIS-NM from SN tissue as it was necessary to remove non-specifically associated proteins. A preliminary study reported that TIS-NM isolated without proteinase K contained a higher percentage of extracellular and nuclear proteins than TIS-NM isolated with proteinase K, indicating a higher contamination by non-specific proteins during the isolation procedure.^29^ Indeed, the isolation of NM pigment without proteinase K generates macroaggregates difficult to purify. These macroaggregates could contain proteins originating from cytosol, other organelles and tissue compartments which interact and bind to NM pigment and NM-conjugated proteins during isolation, that may form S–S bridges and other means of conjugation. In this case, this kind of sample would contain proteins not related to the NM-containing organelle. This procedure and its rationale are described in detail in previous studies (Methods).^3,9^ We elected to prepare the TIS-NM samples with proteinase K as this type of sample was used in several previous studies, and so necessary for comparison of the present data with previous reports.^3,9,28^

Finally, to overcome the above experimental limitations, we further analyzed proteins in ORG-NM samples, i.e., the NM pigment isolated from ORG samples. This type of NM was purified by disrupting and eliminating the membranes and the soluble portion from ORG samples, in order to study the protein components strictly associated with NM pigment, without exposure to contaminants from other intraneuronal components.

This experimental design was in our opinion the best approach to exclude contaminating proteins. In addition, this approach avoided loss of some proteins of the NM-containing organelle, a central aspect of this study, beyond preventing contamination.

We then compared the three sets of proteins observed in the following preparations (Supplementary Fig. 1): two independent samples of ORG, two independent samples of TIS-NM, and two independent samples of ORG-NM (Methods). ORG, TIS-NM, and ORG-NM samples were analyzed by a total of 15 LC-MS analyses. As a result, 1020 proteins were identified and a group of 293 was selected as representative of the samples based on their amount (Table 1), as estimated from the number of spectral count (SpC), defined as the sum of all peptides of a single protein observed in the mass spectrum. The threshold for inclusion in the list of representative proteins was two or more SpC (i.e., peptides) for each protein as average value in at least one of the three types of samples.

**Table 1:** Summary of the proteomic analysis

	ORG (*n* = 2)	TIS-NM (*n* = 2)	ORG-NM (*n* = 2)	Overall
Number of proteins	563	407	220	1020
Number of SpC for all 1020 proteins	4229	7743	2023	13,995
Representative proteins (with SpC ≥ 2 as average value)	174 [164]	130 [116]	125 [101]	293
Number of SpC for the group of 293 representative proteins	3792 [3778]	7439 [7420]	1926 [1898]	13,157 [13,096]

Summary of proteomic analyses of different types of samples (ORG, TIS-NM and ORG-NM), calculated from Supplementary Data 1. Each type of sample was prepared in duplicate and then analyzed by multiple LC-MS analyses. For details of subjects and preparation of samples for LC-MS analysis of proteins, see Methods. The first two rows show the number of proteins and their number of SpC for each kind of sample. Among the group of overall proteins detected in these samples (1020 by 13,995 SpC), representative proteins were selected on the basis of their SpC, defined as the sum of all peptides of a single protein observed in the mass spectrum. We have classified a protein as representative in one type of sample, if detected by SpC ≥ 2 as average value. In the third row, we report for each type of sample the following values: (i) in brackets, the number of proteins detected as representative (with SpC ≥ 2) uniquely in that type of sample; (ii) without brackets, the number of representative proteins in that type of sample plus those identified as non-representative (with SpC < 2) in that specific sample but listed as representative (with SpC ≥ 2) in at least one of the other type of samples (e.g., a protein that was detected as non-representative in TIS-NM but as representative in ORG or ORG-NM samples would be included in the count for TIS-NM). The fourth row of the table shows the number of SpC detected for the group of 293 representative proteins described in the third row, and in particular: (i) in brackets, the total number of SpC detected for representative proteins (with SpC ≥ 2) uniquely found in that type of sample (e.g., 3778 is the number of SpC for 164 proteins uniquely detected in ORG sample); (ii) without brackets, the total number of SpC of representative proteins plus the SpC of proteins identified as non-representative (with SpC < 2) in that specific sample but listed as representative (with SpC ≥ 2) in at least one of the other type of samples (e.g., 7439 is the number of SpC for 130 proteins detected in TIS-NM sample, including SpC of proteins uniquely found in TIS-NM plus those of proteins detected as non-representative in TIS-NM but as representative in ORG or ORG-NM samples). The “Overall” column represents the overall number of proteins and peptides detected in all analyzed samples (i.e., 293 is the number of all representative proteins detected in ORG, TIS-NM, and ORG-NM, considering only one time a protein present in two or more samples)

**Fig. 2:**
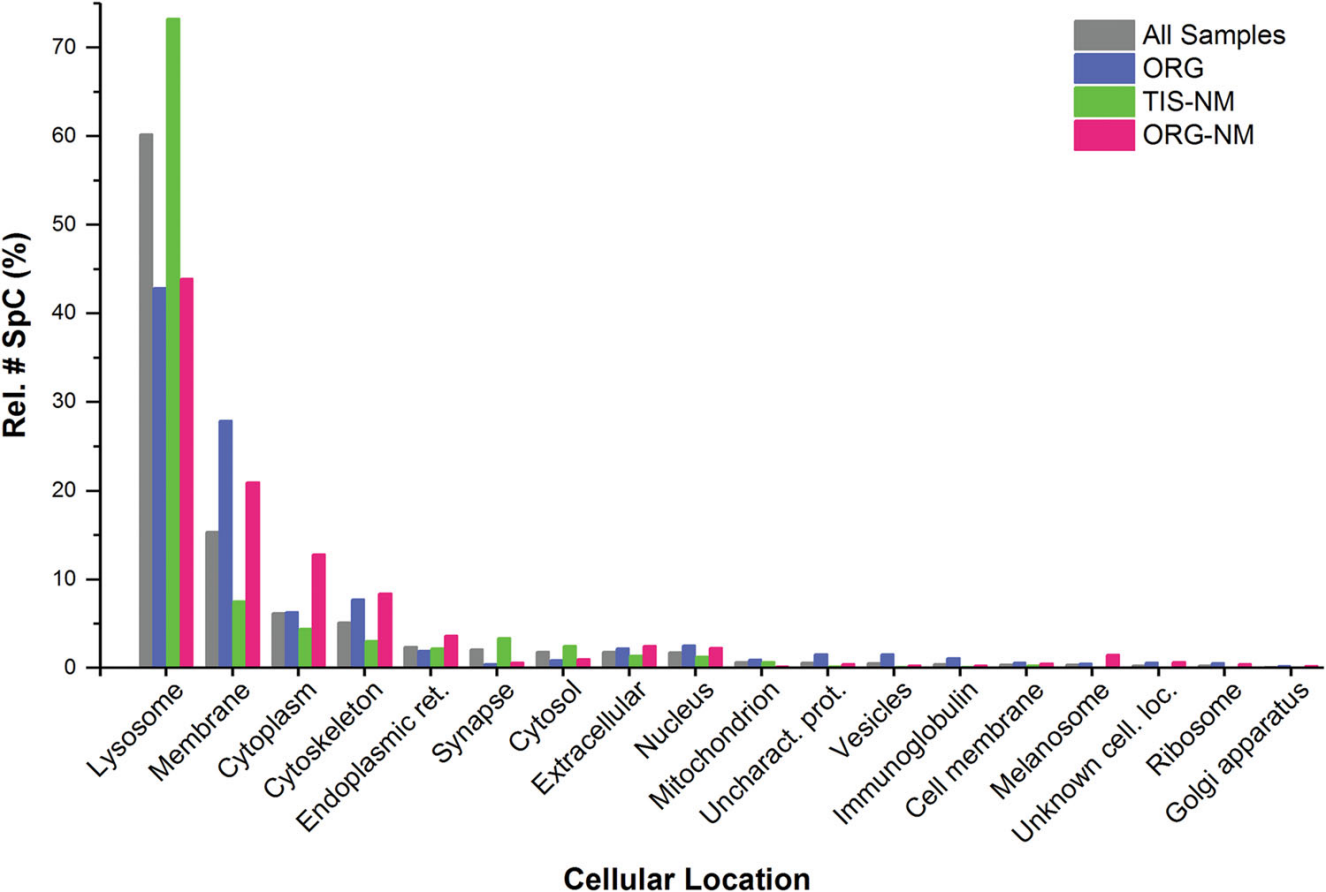
Histogram of cellular distribution of the 293 representative proteins found in all analyzed samples shown as relative number of SpC vs. cellular compartments. For details of subjects and preparation of samples for LC-MS analysis of proteins, see Methods. Some proteins may have multiple cellular locations: for each protein the most typical and representative cellular location was assigned. The different types of samples are represented by different colors (ORG, TIS-NM, and ORG-NM) and gray bars refer to overall representative proteins considered as a single data set (indicated with “All Samples”). The “Rel. # SpC (%)” is the total number of SpC for a specific class of proteins (i.e., lysosomal) referred to the overall number of SpC of representative proteins in each sample: this value represents the relative abundance of a particular class of proteins in one sample (see also Supplementary Table 1). The term “Vesicles” refers to vesicle trafficking, including proteins involved in vesicular transport, fusion, etc. The category “Unknown cell location” consists of proteins for which a cellular location was still unclear, while the class “Uncharacterized proteins” comprises proteins for which, at the moment of data analyses, a complete characterization and/or role was missing

The cellular distribution of the 293 representative proteins is represented in Fig. 2 (for details refer to Supplementary Table 1; Supplementary Data 1), showing that 34 proteins, detected by ∼60 % of the number of SpC in all samples (7916 of 13,157 overall number of SpC for representative proteins), were lysosomal proteins. We can speculate that if all the protein classes were equally represented in our samples, the 34 lysosomal proteins among the 293 representative proteins (Supplementary Table 1) would be represented by ∼1527 SpC and not by 7916 SpC, as we observed. With this assumption, we estimate that the lysosomal class is >5-fold enriched in our samples. This is a striking evidence of lysosomal protein overrepresentation.

**Fig. 3:**
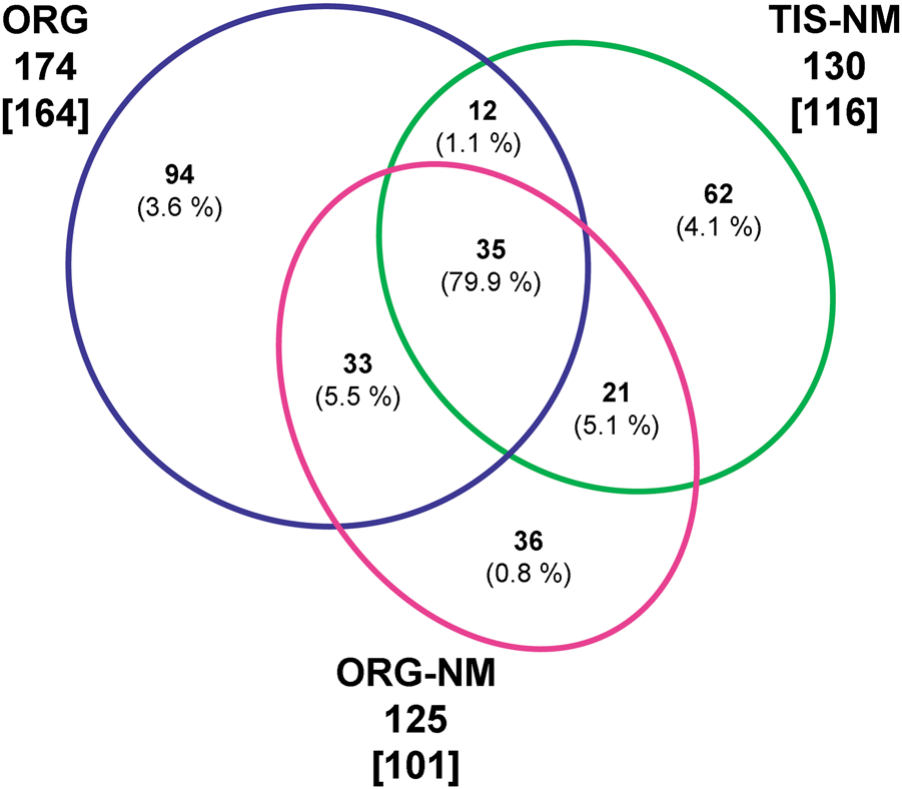
Area-proportional Euler diagram of the 293 representative proteins (detected by SpC ≥ 2 as average value in at least one of the three types of samples) identified in ORG, TIS-NM, or ORG-NM. For details of subjects and preparation of samples for LC-MS analysis of proteins, see Methods. The diagram was calculated using the EulerAPE tool (Methods)^138^ and by using NCBI accession (GI number). Outside the diagram we report for each type of sample the following values: (i) in brackets, the number of proteins detected as representative (with SpC ≥ 2) uniquely in that type of sample, as reported in Table 1; (ii) without brackets, the number of representative proteins plus those identified as non-representative (with SpC < 2) in that specific sample but listed as representative (with SpC ≥ 2) in at least one of the other type of samples (e.g., a protein that was detected as non-representative in ORG sample but as representative in TIS-NM would be included in the count for ORG). Numbers in non-overlapping areas of circles report the representative proteins found uniquely in that type of sample. The overlapping areas correspond to proteins shared by two or three different types of samples: e.g., a protein detected in all samples but as representative only in ORG would be included in the overlapping area of 35 proteins shared between ORG, TIS-NM, and ORG-NM. Percentages in parentheses represent the ratio between the total number of SpC of proteins belonging to one area of the diagram and the overall number of SpC of representative proteins detected in all samples. The highest percentage value is located in the area shared between all three types of samples. The detailed list of proteins is reported in Supplementary Data 1

An Euler diagram in Fig. 3 shows the distribution of the 293 representative proteins among different samples, as described in Table 1. The ORG sample contains the highest number of proteins and shares 80 proteins with the other two samples. TIS-NM and ORG-NM, representing two different preparations of NM pigment, contain fewer proteins. TIS-NM shares 68 proteins with ORG and ORG-NM samples, while ORG-NM shares 89 proteins with ORG and TIS-NM samples. A portion of representative proteins was uniquely identified in each type of sample (94 proteins in ORG, 62 proteins in TIS-NM, and 36 proteins in ORG-NM), but these were present in very small quantities (<10 % of the overall number of SpC detected for representative proteins); interestingly, 35 proteins were commonly detected in all three samples, and these shared proteins were present in high quantities (∼80 % of the overall number of SpC detected for representative proteins; see data and discussion in the legends of Fig. 3 and Table 1, and details in following sections).

### Proteins found in NM-containing organelles

The ORG samples were obtained directly from fresh SN tissue, without freezing and after soft homogenization/centrifugation procedures in order to preserve the original structure and composition of these organelles. Transmission electron microscopy confirmed that the contents of the purified organelles were mainly intact, with preserved lipid membranes and lipid bodies that were morphologically identical to those observed in slices of SN tissues. Importantly, low magnification images demonstrated the absence of other cellular contaminants (Fig. 1d).

A group of 164 proteins was identified with SpC ≥ 2 exclusively in ORG sample (Table 1; Supplementary Data 1), while an additional ten proteins were identified with SpC < 2 in ORG but detected with SpC ≥ 2 in TIS-NM or ORG-NM samples. Then, we are considering that the number of representative proteins in ORG sample is 174 (Table 1; Fig. 3; Supplementary Table 1).These data revealed that ORG is characterized by a large group of lysosomal proteins (25 proteins, ~43 % rel. # SpC), with far fewer proteins from other organelles or cytosol (Fig. 2; Supplementary Table 1). In addition to the set of soluble lysosomal proteins observed in all three types of samples (proteases, esterases, sulfatases, glycosidases, hydrolases, and other lysosomal proteins), here we identified typical lysosomal membrane proteins including lysosome membrane protein 2 (SCARB2), CD63 antigen, type 1 phosphatidylinositol 4,5-bisphosphate 4-phosphatase (only in the ORG sample) and some functional subunits of the lysosomal V-type proton ATPase (in ORG sample one subunit as representative while other two subunits in very low amounts and categorized as non-representative proteins), but not lysosome-associated membrane glycoprotein 1 (LAMP1) or lysosome-associated membrane glycoprotein 2 (LAMP2).

**Fig. 4:**
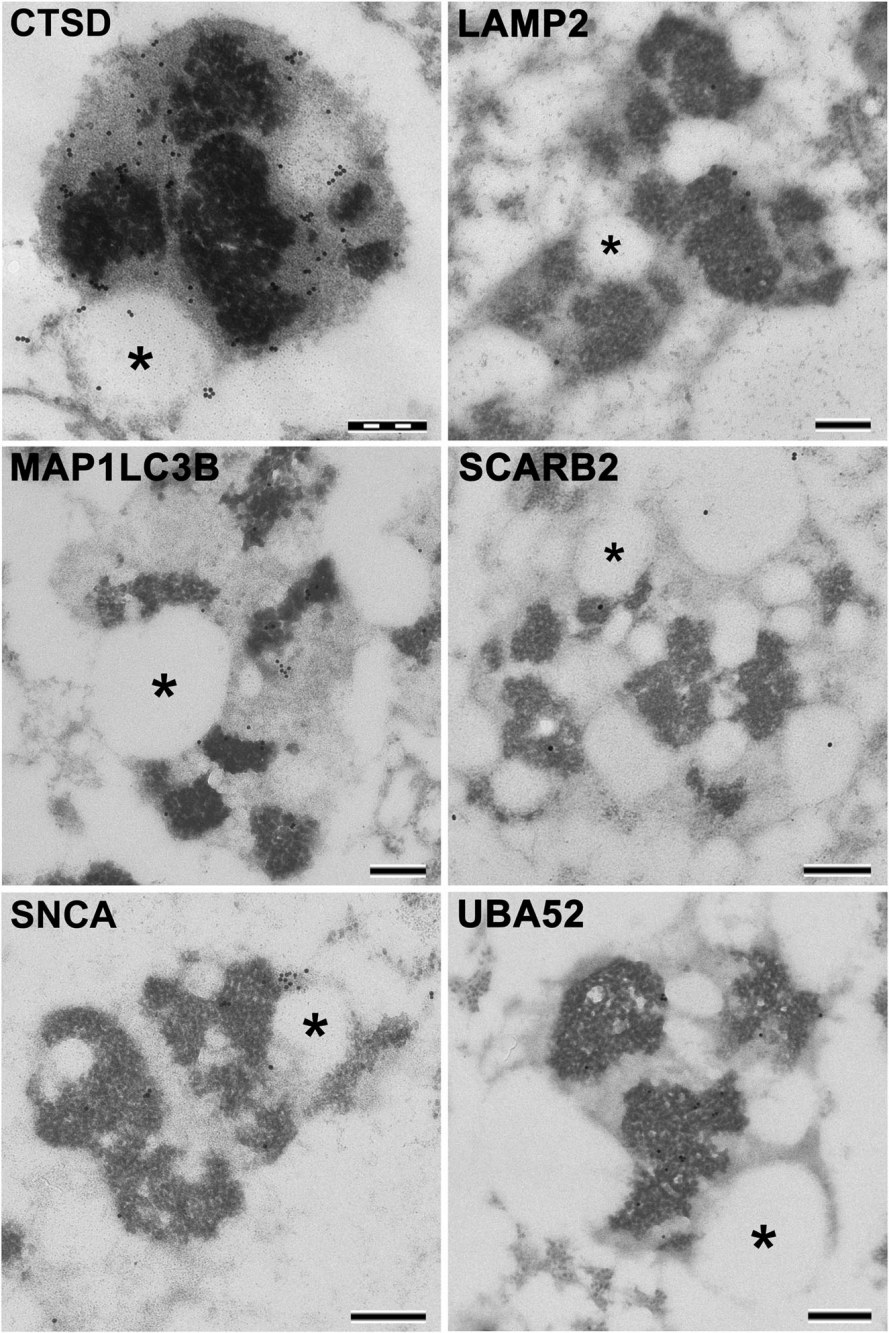
IEM of SN from healthy aged subjects for selected proteins. For number of IEM experiments, see Methods. CTSD (73 y.o.; gold particles = 20 nm). LAMP2 (86 y.o.; gold particles = 20 nm). MAP1LC3B (69 y.o.; gold particles = 15 nm). SCARB2 (69 y.o.; gold particles = 15 nm). SNCA (63 y.o.; gold particles = 15 nm). UBA52 (63 y.o.; gold particles = 15 nm). Lipid bodies are indicated by asterisks. NM pigment of the NM-containing organelles appears as black and electron dense granular aggregates. Scale bar in each panel = 250 nm

**Fig. 5:**
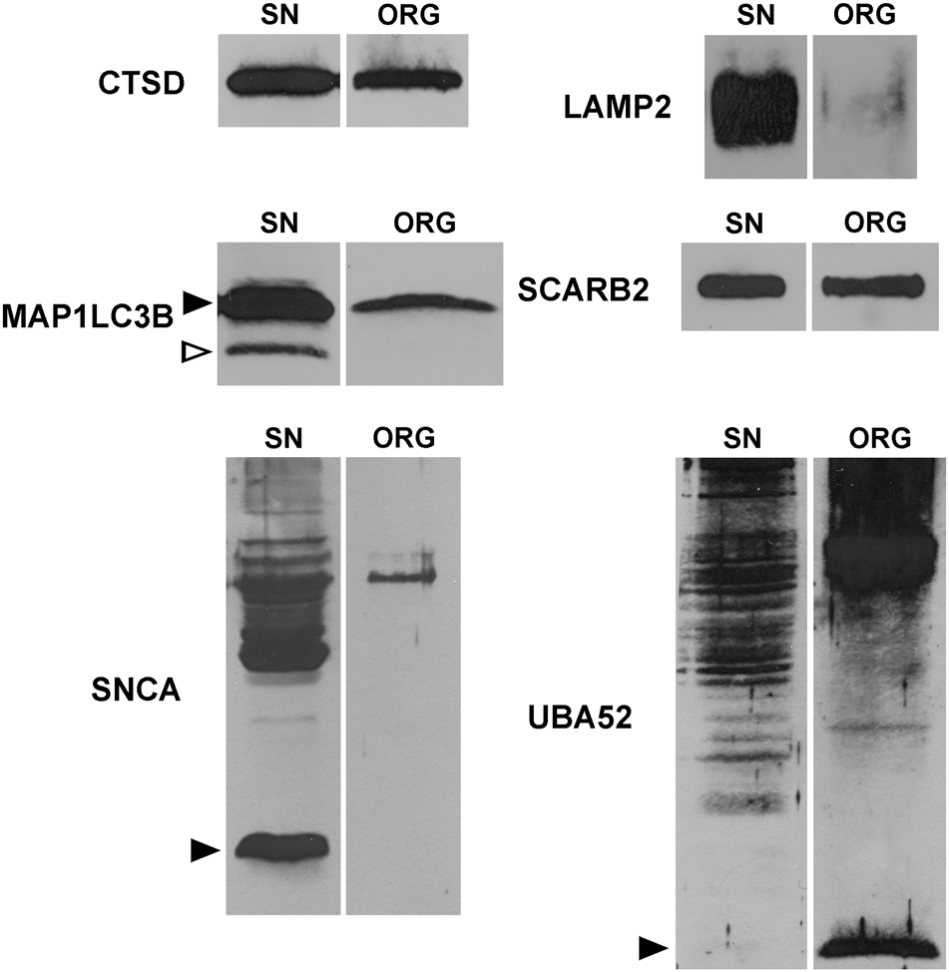
WB (for proteins detected by IEM in Fig. 4) performed on SN tissue lysates and on ORG samples. For number of WB analyses, see Methods. CTSD (protein content ratio SN tissue lysate/ORG = 3.1). The band present in both SN tissue lysate (16 pooled tissues, from 48 to 85 years of age) and ORG sample (isolated from one subject, 81 y.o.) corresponds to the mature CTSD heavy chain which is highly enriched in ORG sample, considering that the total protein content in ORG was 3.1-fold lower than that of SN tissue lysate. LAMP2 (protein content ratio SN tissue lysate/ORG = 1.0). LAMP2 protein was lightly present in ORG sample (isolated from one subject, 83 y.o.), while in SN tissue lysate (13 pooled tissues, from 62 to 86 years of age) this protein is largely expressed. The antibody used here recognizes all three LAMP2 isoforms. MAP1LC3B (protein content ratio SN tissue lysate/ORG = 1.8). The black arrowhead indicates the MAP1LC3B-I form, which was more prevalent in SN tissue lysate (five pooled tissues, from 73 to 85 years of age) than the MAP1LC3B-II form (empty arrowhead indicating the phosphatidylethanolamine conjugated form). In ORG sample (isolated from one subject, 77 y.o.), the MAP1LC3B-I form was abundant while MAP1LC3B-II form was undetectable. SCARB2 (protein content ratio SN tissue lysate/ORG = 2.3). Here we note an enrichment of SCARB2 in ORG sample (isolated from one subject, 77 y.o.) if compared to SN tissue lysate (five pooled tissues, from 73 to 85 years of age), considering that the total protein content in ORG was 2.3-fold lower than that of SN tissue lysate. SNCA (protein content ratio SN tissue lysate/ORG = 3.4). The black arrowhead indicates the soluble-monomeric form of SNCA which is clearly visible in SN tissue lysate (nine pooled tissues, from 67 to 85 years of age) while undetectable in ORG sample (isolated from one subject, 66 y.o.). Other bands at higher molecular weight are present in SN tissue lysate, corresponding to fibrils and aggregates with possible modifications. In the ORG sample, two main bands are clearly visible corresponding to some aggregated/modified forms of SNCA (at ~50 and ~58 kDa) which are present also in SN tissue lysate. UBA52 (protein content ratio SN tissue lysate/ORG = 2.7). The black arrowhead indicates the free ubiquitin that is scarcely visible in SN tissue lysate (eight pooled tissues, from 62 to 89 years of age), but abundant in the ORG sample (isolated from one subject, 66 y.o.). The WB also reveals the presence of large number of immunoreactive high molecular weight bands corresponding to high amounts of poly-ubiquitinated proteins, both in SN tissue lysate and highly enriched in the ORG sample, although the total protein content in ORG was 2.7-fold lower than that of SN tissue lysate

Due to the difficulties in preserving ORG membranes during their isolation and related problems in LC-MS detection of transmembrane proteins, we also performed WB and IEM experiments to investigate lysosomal features of these organelles. We confirmed the presence of SCARB2 and V-type proton ATPase subunit B, brain isoform (ATP6V1B2), a subunit of the lysosomal V-type proton ATPase, in NM-containing organelles by WB on ORG samples and by IEM in SN sections (Figs. 4 and 5; Supplementary Fig. 2, 3). Likewise, both LAMP1 and LAMP2, while undetected by LC-MS in ORG samples, were observed by WB and IEM (Figs. 4 and 5; Supplementary Fig. 2, 3).

Some non-lysosomal proteins were also detected, most of which are classified as cytoskeletal and cytoplasmatic proteins. In addition to typical tubulin chains (mainly beta chains), we found tubulin polymerization-promoting protein, which is involved in protein aggregation, inclusion bodies formation and neurodegeneration;^30^ we also detected heat shock protein HSP 90-alpha and alpha-crystallin B chain, which has been reported as a component of Lewy bodies and has been characterized as a chaperone.^31^ Additional cytoskeletal proteins included for example the microtubule-associated protein tau and microtubule-associated protein 6, which play roles in microtubule stability and are implicated in neurodegenerative mechanisms.^32,33^

As a potentially important clue in PD pathogenesis,^34,35^ we also detected alpha-synuclein (SNCA) exclusively in ORG samples. We confirmed this result by WB experiments on ORG samples and IEM experiments on SN tissue slices, observing SNCA signals mainly in the NM pigment and rarely in lipid bodies (Figs. 4 and 5).

We also found major histocompatibility complex, class I (HLA) in ORG samples, consistent with our recent report of the first identification of this protein in adult neurons.^20^ The identification of HLA was obtained by matching experimental spectra to peptide sequences in specific databases for HLA (Supplementary Data 2). The presence of the antigen presenting protein HLA was confirmed with IEM on SN tissue, indicating a high accumulation of HLA on NM granules of the SN (Supplementary Fig. 4).

Interestingly, some additional proteins identified only in ORG sample were dynein heavy chain 12, axonemal and some ras-related proteins (RAB8B, RAB14, and RAB33B as representative proteins, while RAB2A, RAB5C, and RAB8A were present in low amounts and categorized as non-representative), each of which are involved in intracellular vesicle trafficking.

### Proteins found in NM pigment isolated from SN tissue

The TIS-NM samples were obtained from pooled SN tissues using the purification procedure adopted for previous chemical and structural investigations on NM pigment.^3,9,28^ Despite the chemical treatments (i.e., high-salt solutions, sodium dodecyl sulfate, methanol, and hexane), repeated washings and proteinase K digestions, proteomic analysis resulted in the identification of 116 proteins with SpC ≥ 2 uniquely in TIS-NM (Table 1; Supplementary Data 1). However, an additional 14 proteins with SpC < 2 in TIS-NM were found with SpC ≥ 2 in ORG or ORG-NM samples: therefore, we estimate 130 to be the number of representative proteins in TIS-NM sample (Table 1; Fig. 3; Supplementary Table 1). The most highly represented and abundant class of proteins was again lysosomal, even more so than in ORG samples. We detected 26 lysosomal proteins representing ~73 % of rel. # SpC in this sample (Fig. 2; Supplementary Table 1). Among these proteins, high quantities of typical lysosomal enzymes were found, and these were also identified in ORG and ORG-NM samples. However, some lysosomal proteins were detected exclusively in TIS-NM samples (e.g., epididymal secretory protein E1, fatty acid synthase, cathepsin L1, lysosomal alpha-mannosidase, and ribonuclease T2). Only one lysosomal membrane protein, the transmembrane protein 106B, was identified exclusively in the TIS-NM sample.

Non-lysosomal proteins found in TIS-NM included ferritins, mostly ferritin light chain (FTL) in contrast to ferritin heavy chain (FTH1), heat shock protein HSP 90-alpha, and glyceraldehyde-3-phosphate dehydrogenase. HLA was also detected in this sample (Supplementary Data 2). By means of LC-MS analysis, we found exclusively in this sample the mature chain of ATP synthase F(0) complex subunit C3, mitochondrial (ATP5G3), the major storage material accumulated in ceroid lipofuscinosis,^36^ as well as cerebellin-2, the PD-associated protein DJ-1 and protein disulfide-isomerase A3. Additionally superoxide dismutase [Cu–Zn] was detected in TIS-NM and also as a fragment in ORG sample. The ATP synthase F(0) complex subunit C1, mitochondrial (ATP5G1) was also detected by WB in ORG samples; its presence was also confirmed by IEM, indicating that is localized in the NM-containing organelles, where it is mainly bound to the NM pigment. The antibody used in these experiments is expected to recognize the three mature chains of proteins, namely ATP5G1, ATP synthase F(0) complex subunit C2, mitochondrial (ATP5G2) and ATP5G3, which are identical and encoded by three different genes (Supplementary Fig. 2, 3).

### Proteins found in NM pigment isolated from organelles

In order to study the protein matrix associated with the NM pigment inside organelles and considering that aspecific proteins might interact with NM during the isolation of TIS-NM from SN tissues, we isolated NM pigment directly from ORG samples. The amount of ORG-NM sample was lower than the other two types of samples (ORG-NM < ORG < TIS-NM), as it was prepared from the ORG sample; TIS-NM was isolated processing several SN pooled tissues, while ORG and ORG-NM samples were prepared from one or occasionally two SN tissues (Methods). This is clearly shown by the number of SpC detected in each sample (Table 1). A group of 101 proteins was detected with SpC ≥ 2 exclusively in ORG-NM (Table 1; Supplementary Data 1), while an additional 24 proteins were identified in ORG-NM with SpC < 2 but detected with SpC ≥ 2 in ORG or TIS-NM samples. Therefore, we estimate the number of representative proteins in ORG-NM sample to be 125 (Table 1; Fig. 3; Supplementary Table 1); the majority (89 proteins) were also detected in ORG and TIS-NM samples (Fig. 3). Proteins identified only in ORG-NM sample (36 proteins) were found in very low amounts (<1 % rel. # SpC) (Fig. 3), thus indicating that almost no contaminants affected the analysis of this type of sample.

As observed in ORG and TIS-NM, lysosomal proteins were also prevalent in this sample (23 proteins; ~44 % rel. # SpC) (Fig. 2; Supplementary Table 1). Moreover, lysosomal membrane proteins (i.e., SCARB2 and CD63 antigen) and other cytoskeletal and cytoplasmatic proteins (i.e., alpha-crystallin B chain, tubulin polymerization-promoting protein, microtubule-associated protein tau, and low amounts of microtubule-associated protein 6) found in ORG samples were observed in ORG-NM as well. Again, LAMP2 was not detected in this sample by LC-MS, while LAMP1 was identified at very low levels and categorized as a non-representative protein. These data suggest that thermal shock treatments to purify NM pigment from organelles do not completely disrupt the organelle. It is possible that some membrane portions are strongly connected to the contents of the organelle and prevent the leakage of some proteins. Indeed, membranous, cytoskeletal and cytoplasmic proteins were still found in the analysis of ORG-NM sample. As observed in ORG and in TIS-NM samples, HLA was also detected in this sample (Supplementary Data 2).

Similarly, ORG-NM samples share several proteins with TIS-NM (Fig. 3): e.g., the lysosomal prosaposin, heat shock protein HSP 90-alpha (here detected in low amounts, but present as representative also in ORG and TIS-NM), glyceraldehyde-3-phosphate dehydrogenase, some peptides of ferritins (again mostly FTL if compared to FTH1), and the PD-associated protein ubiquitin carboxyl-terminal hydrolase isozyme L1 (here as representative, while in TIS-NM it was in low amounts and categorized as a non-representative protein). ORG-NM and TIS-NM are analogous samples since they are both isolated NM pigment. However, the amount of ORG-NM sample is normally lower than that of TIS-NM, while the ORG sample is the intact organelle containing the NM pigment. Due to the identification by LC-MS of FTL and FTH1 in both ORG-NM and TIS-NM samples but not in ORG samples, IEM and WB experiments were performed. IEM confirmed the presence of FTL and to a lesser extent FTH1 in NM-containing organelles of the SN tissue. The accumulation of this iron-storage protein was confirmed by WB analyses that detected low levels of FTL but failed to detect FTH1 in ORG samples (Supplementary Fig. 2, 3). This may be due to chains of iron hydroxides that formed bridges connecting NM and partially degraded ferritins, so that this protein was bound to NM and was not present as a separate molecule.

Examples of proteins identified as representative only in ORG-NM include phospholipid hydroperoxide glutathione peroxidase, mitochondrial, and lysosomal acid phosphatase.

### Proteins detected in all three types of samples

A common group of proteins was identified in all types of samples, corresponding to ~80 % of overall SpC detected for 293 representative proteins (Fig. 3; Table 2).

**Table 2:** Representative proteins commonly detected by LC-MS in all analyzed samples

NCBI accession (GI number)	UniProt accession number	Protein name	Gene name	Cellular location	All samples	
					Total # SpC	Average # SpC
193785841	P61981	14-3-3 protein gamma	*YWHAG*	Cytoplasm	13	2
21536286	P12277	Creatine kinase B-type	*CKB*	Cytoplasm	41	6
4503979	P14136	Glial fibrillary acidic protein	*GFAP*	Cytoplasm	124	14
21735492	Q9UD71	Protein phosphatase 1 regulatory subunit 1B	*PPP1R1B*	Cytoplasm	28	3
32526901	Q8WYA0	Intraflagellar transport protein 81 homolog	*IFT81*	Cytoskeleton	17	2
105990539	P07196	Neurofilament light polypeptide	*NEFL*	Cytoskeleton	10	2
2119276	Q6LC01	Tubulin beta chain (fragment)	*N/A*	Cytoskeleton	126	11
4507729	Q13885	Tubulin beta-2A chain	*TUBB2A*	Cytoskeleton	22	3
21361322	P04350	Tubulin beta-4A chain	*TUBB4A*	Cytoskeleton	60	8
60729665	Q6Y288	Beta-1,3-glucosyltransferase	*B3GLCT*	Endoplasmic reticulum	17	2
1575347	Q8IV08	Phospholipase D3	*PLD3*	Endoplasmic reticulum	266	19
4502163	P05090	Apolipoprotein D	*APOD*	Extracellular	74	10
8247915	Q13510	Acid ceramidase	*ASAH1*	Lysosome	225	17
825628	P15848	Arylsulfatase B	*ARSB*	Lysosome	53	4
4503139	P07858	Cathepsin B	*CTSB*	Lysosome	1058	63
3929733	Q6LAF9	Cathepsin B (fragment)	*N/A*	Lysosome	88	10
4503143	P07339	Cathepsin D	*CTSD*	Lysosome	343	24
22538442	Q9UBR2	Cathepsin Z	*CTSZ*	Lysosome	169	11
4503987	Q92820	Gamma-glutamyl hydrolase	*GGH*	Lysosome	1003	62
4557659	P22304	Iduronate 2-sulfatase	*IDS*	Lysosome	95	8
126590	P10253	Lysosomal alpha-glucosidase	*GAA*	Lysosome	37	3
4826940	P42785	Lysosomal Pro-X carboxypeptidase	*PRCP*	Lysosome	91	7
24475586	Q9UM22	Mammalian ependymin-related protein 1	*EPDR1*	Lysosome	1489	88
4503899	P34059	*N*-acetylgalactosamine-6-sulfatase	*GALNS*	Lysosome	26	2
4506919	P51688	*N*-sulphoglucosamine sulphohydrolase	*SGSH*	Lysosome	47	4
4506031	P50897	Palmitoyl-protein thioesterase 1	*PPT1*	Lysosome	287	19
27734917	Q8NHP8	Putative phospholipase B-like 2	*PLBD2*	Lysosome	57	4
6808138	Q9HAT2	Sialate *O*-acetylesterase	*SIAE*	Lysosome	2043	124
2408232	O14773	Tripeptidyl-peptidase 1	*TPP1*	Lysosome	428	26
4505405	Q14956	Transmembrane glycoprotein NMB	*GPNMB*	Melanosome	49	11
68509930	P02686	Myelin basic protein	*MBP*	Membrane	1867	125
5912201	Q86XK2	F-box only protein 11	*FBXO11*	Nucleus	21	4
31542868	P13807	Glycogen [starch] synthase, muscle	*GYS1*	Nucleus	6	1
4506645	P63173	60S ribosomal protein L38	*RPL38*	Ribosome	8	2
4757922	P23435	Cerebellin-1	*CBLN1*	Synapse	226	14

List of representative proteins commonly detected by LC-MS analyses in all samples. In the table for each protein, NCBI accession (GI number), UniProt accession number, protein and gene name, cellular location, and few details of LC-MS analyses corresponding to all samples are reported. The column “All Samples” refers to overall representative proteins commonly present in all three types of samples which are considered as a single data set. Note that some proteins may have multiple cellular locations: for each protein the most typical and representative cellular location was assigned. A protein eligible to be inserted in this list must be detected in all samples, and classified as representative (detected by SpC ≥ 2 as average value) in at least one of the three types of samples. This list describes the 35 proteins contained in the central overlapping area of Euler diagram in Fig. 3. For full details see Supplementary Data 1. Two ubiquitin-related proteins (UBC and UBA52) and heat shock protein HSP 90-alpha, although present in the three types of samples, were not included in the group of proteins detected in all three types of samples (in this list and in the central overlapping area of Euler diagram in Fig. 3, containing 35 proteins) because they were identified with different GI accession numbers in different samples. Note that the Euler diagram calculation was performed using NCBI accession (GI number) only. Nevertheless, the above mentioned ubiquitin-related proteins and heat shock protein HSP 90-alpha, considered as unique proteins, were detected in all three samples

Within the set of lysosomal proteins detected in all three types of samples (17 entries in Table 2), were peptidases (tripeptidyl-peptidase 1, gamma-glutamyl hydrolase and lysosomal Pro-X carboxypeptidase), proteases [cathepsin B, cathepsin Z and cathepsin D (CTSD)], esterases (sialate *O*-acetylesterase and palmitoyl-protein thioesterase 1), sulfatases (iduronate 2-sulfatase, *N*-sulphoglucosamine sulphohydrolase, arylsulfatase B and *N*-acetylgalactosamine-6-sulfatase), glycosidases (lysosomal alpha-glucosidase), lipid hydrolases (acid ceramidase), and other lysosomal proteins [mammalian ependymin-related protein 1 and putative phospholipase B-like 2 (PLBD2)]. CTSD, a classical lysosomal marker, was detected in ORG samples by WB as heavy chain mature form and was confirmed by IEM as present abundantly in the NM-containing organelles (Figs. 4 and 5). We confirmed by IEM and WB (Supplementary Fig. 2, 3) the presence of the recently described PLBD2.^37,38^ In addition, we detected by LC-MS phospholipase D3, apolipoprotein D (APOD), cerebellin-1, transmembrane glycoprotein NMB (GPNMB), F-box only protein 11, heat shock protein HSP 90-alpha, and two ubiquitin-related proteins, namely polyubiquitin-C (UBC) and ubiquitin-60S ribosomal protein L40 (UBA52). HLA peptides associated with NM were identified by LC-MS analyses in all samples isolated from human SN (Supplementary Data 2). In each type of sample we also found protein phosphatase 1 regulatory subunit 1B, which potently inhibits protein phosphatase-1.^39^ Moreover, various tubulins (mainly beta chains) and other abundant proteins including glial fibrillary acidic protein, myelin basic protein, and creatine kinase B-type were detected in all three samples (for other proteins see Table 2; Supplementary Data 1). Due to the important roles of APOD, GPNMB, ubiquitins, and HLA, we conducted IEM and WB experiments, which confirmed their localization inside the NM-containing organelles (Figs. 4 and 5; Supplementary Fig. 4, 5, 6).

### Relevant proteins not revealed by mass spectrometry but found by other techniques

Additional IEM and WB studies were performed for some relevant proteins not detected by LC-MS, probably due to instrumental limitations and low abundance. Due to the lysosomal features of the NM-containing organelle and its proposed autophagic origin,^2^ we performed WB and IEM experiments to verify the presence of the autophagic marker microtubule-associated proteins 1A/1B light chain 3B (MAP1LC3B), which was not detected by LC-MS like in previous studies.^23,24^ Here, IEM experiments on SN tissues revealed MAP1LC3B signals mainly engulfed inside NM-containing organelles, on NM pigment, around lipid bodies and sometimes lining their membranes (Fig. 4). In order to confirm the presence of this crucial protein, IEM experiments were also repeated using a different antibody, confirming the specific accumulation of MAP1LC3B inside NM-containing organelles (Supplementary Fig. 4). In addition to IEM findings, WB analyses of ORG samples demonstrated the presence of MAP1LC3B, thus confirming the autophagic nature of NM-containing organelle. Specifically, WB analyses demonstrated mainly the MAP1LC3B-I form, while the MAP1LC3B-II form was undetectable in ORG samples (Fig. 5). Another important autophagy-related protein, the autophagic adaptor sequestosome-1 (SQSTM1), was not detected by LC-MS here and in previous studies,^23,24^ but was found by IEM and WB analyses (Supplementary Fig. 5, 6).

Moreover, due to the presence of some Ras-related proteins, we confirmed the presence of Ras-related protein Rab-5A (RAB5A), which is of interest due to its involvement in retrograde axonal endosomal transport,^40^ by IEM and WB (Supplementary Fig. 5, 6) but not by LC-MS.

### Lipid bodies contain dolichols involved in NM synthesis and typical membrane lipids

Within NM-containing organelles, electron micrographs demonstrated the presence of membrane-bound lipid bodies, generally ranging from 200 to 500 nm, sometimes reaching sizes as large as 1 µm, with some smaller lipid bodies (50–100 nm) entrapped in the NM regions of the organelle (Fig. 1a–c). Conventional electron microscopy on *post mortem* SN tissues does not clearly reveal if these lipid bodies are surrounded by membranes formed of normal bilayer or single layer.

**Fig. 6:**
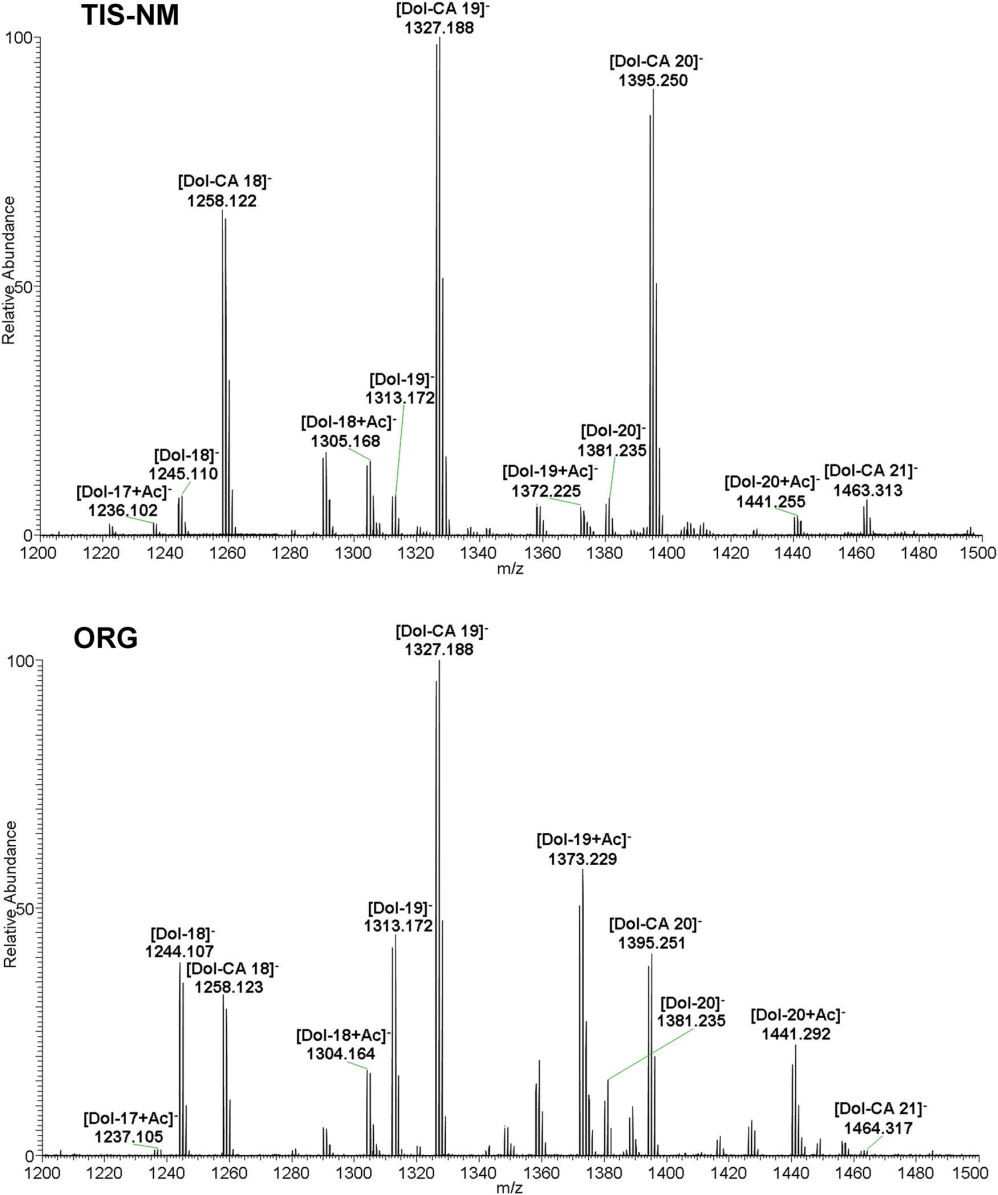
LC-MS analysis of lipids isolated from TIS-NM and ORG samples. The TIS-NM sample here represented was isolated from a pool of seven subjects (from 71 to 85 years of age), while the ORG sample was isolated from two pooled subjects (74 and 89 y.o.). Mass spectra (averaged mass spectra, range 1200–1500 m/z) demonstrate the presence of dolichols species in both samples. We highlight the series of singly charged ions with different chain lengths, corresponding to dolichols with terminal hydroxyl group, their oxidized derivative dolichoic acids, and acetate adducts of dolichols species. Both spectra selectively show dolichol species with chain lengths ranging from 17 to 21 isoprene units, although few dolichol species with lower and higher number of isoprene units were found in lipid extracts from both samples (Results). Abbreviations used in the figure: Dol, dolichol; Dol-Ac, dolichol acetate; Dol-CA, dolichoic acid

**Fig. 7:**
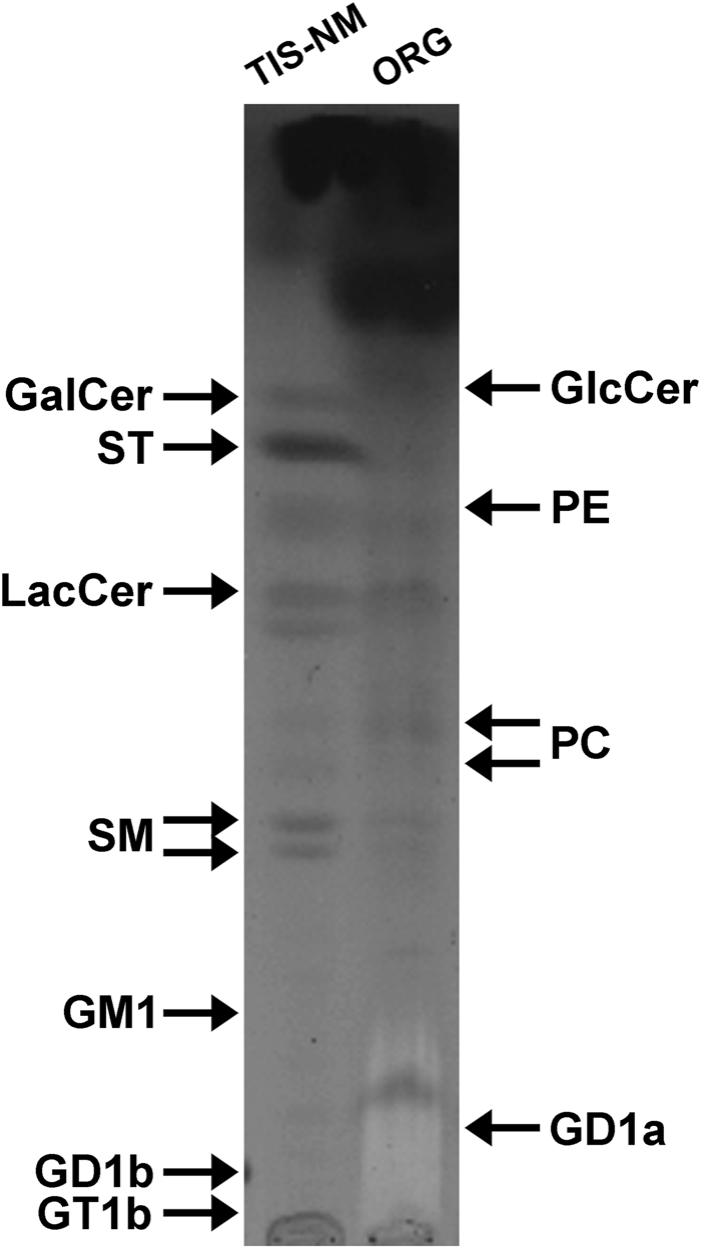
High performance TLC analysis of total lipid extracts obtained from TIS-NM and ORG samples. The TIS-NM sample here represented was isolated from a pool of four subjects (from 62 to 86 years of age), while lipids of from three ORG samples (each isolated from three different subjects, respectively 62, 61 and 77 y.o.) were pooled before loading onto the TLC plates. After separation, lipids were detected by spraying the TLC plate with anisaldehyde. In TIS-NM sample the intense spot at the solvent front likely corresponds to dolichols and dolichoic acids, as confirmed by LC-MS (Fig. 6). The content of sphingomyelin, galactosylceramide, sulfatides (typical myelin lipids), and lactosylceramide is higher than phosphatidylethanolamine and phosphatidylcholine (glycerophospholipids). In the ORG samples, the main components are again dolichols and dolichoic acids at the solvent front. In this sample there are comparable amounts of sphingolipids (lactosylceramide and sphingomyelin) and glycerophospholipids. The arrows at the margin of the image indicate the position of pure standard lipids co-chromatographed with the samples, as described in Methods. Abbreviations used in the figure: GalCer, galactosylceramide; GD1a, GD1b, GM1, GT1b, gangliosides GD1a, GD1b, GM1, GT1b; GlcCer, glucosylceramide; LacCer, lactosylceramide; PC, phosphatidylcholine; PE, phosphatidylethanolamine; SM, sphingomyelin; ST, sulfatides

LC-MS analyses of solvent extracts prepared from both TIS-NM and ORG samples demonstrate that dolichols and dolichoic acids were the major lipid components (Fig. 6). LC-MS analyses of both solvent extracts further revealed the presence of signals corresponding to different glycerophospholipids and sphingolipids (Supplementary Table 2). Among sphingolipids, signals attributable to sphingomyelin, neutral glycolipids (lactosylceramide), sulfatides, and gangliosides (mono-, di-, and tri-sialogangliosides) together with other lipid molecules such as free fatty acids were identified (Supplementary Table 2). Thus, the lipid bodies of ORG and the lipid mixtures adsorbed to TIS-NM contain a wide variety of membrane amphipathic lipids, encompassing both lipids typically enriched in neurons, such as gangliosides, and lipids involved in oligodendrocyte function and myelin formation, such as sphingomyelin and sulfatide.^25,41^ The profile of amphipathic lipids was not identical in TIS-NM and in ORG, in particular as signals attributable to sulfatides were far higher in TIS-NM (Supplementary Table 2), suggesting that some myelin lipids could be adsorbed to TIS-NM during the purification procedure. Indeed, the presence of amphipathic lipids in the lipid bodies from ORG and in lipid mixtures adsorbed to TIS-NM was confirmed by TLC analysis (Fig. 7). In the solvent extracts of the TIS-NM and ORG samples, the amounts of dolichols and dolichoic acids were higher than other lipid molecules identified in both samples, as shown by TLC analysis. In the lipid extracts from both samples, bands co-migrating with sphingomyelin, phosphatidylcholine, lactosylceramide, and phosphatidylethanolamine were identified. In addition, bands corresponding to galactosylceramide and sulfatide (some of the typical myelin lipids)^41^ were clearly visible in the lipid extracts from TIS-NM, particularly sulfatides as also confirmed by LC-MS, but not from ORG samples (Fig. 7). In the ORG sample, there were comparable amounts of some sphingolipids (lactosylceramide and sphingomyelin, a typical myelin lipid) and glycerophospholipids (phosphatidylcholine and phosphatidylethanolamine) (Fig. 7): since in membranes the sphingolipid content is usually lower than that of glycerophospholipids, the relative increase of sphingolipids vs. glycerophospholipids in ORG likely indicates an inhibition of the lysosomal activity inside NM-containing organelles. The presence of mono- and polysialogangliosides was confirmed by cholera toxin staining after sialidase treatment of the aqueous phases obtained from TIS-NM, but not from ORG, likely due to the paucity of the sample (Supplementary Fig. 7). We note that in TIS-NM the membranes and any component of organelles are removed after sodium dodecyl sulfate treatment, but lipids and especially dolichols are still adsorbed into NM structure, as shown after solvent (methanol and hexane) extraction of NM and analysis of these lipids extracts by LC-MS and TLC. The results indicate a selective affinity of NM pigment for some lipids, particularly dolichols and dolichoic acids, as previously reported.^3,9^

The LC-MS results indicate that a high level of dolichols at different molecular weights (with 14–22 isoprene units) and their oxidized derivatives such as dolichoic acids (with 14–21 isoprene units) were present in TIS-NM samples, consistent with previous LC-MS studies on lipids extracts from TIS-NM.^3,42^ This is probably a consequence of membrane disruption during isolation of TIS-NM, so that oxidized dolichols and dolichoic acids present in cytosol, mitochondria and other organelles are released and then adsorbed by NM pigment. However, the presence of dolichols and dolichoic acids was also confirmed by LC-MS in lipid extracts from ORG samples, thus confirming the specific accumulation of this particular class of lipids inside the NM-containing organelles (Fig. 6), mainly in their lipid bodies. Indeed, Fig. 1d shows an electron microscopy image of NM-containing organelles isolated from SN (ORG samples) with many lipid bodies. In addition, the distribution of dolichols and dolichoic acids chain lengths in lipids extracted from ORG samples was similar to that observed in TIS-NM samples: 14–22 isoprene units for dolichols, and 14–21 isoprene units for dolichoic acids. This suggests that artifacts were absent, as the same types of lipids were identified using two different isolation procedures.

No known enzymes involved in dolichol metabolism were observed in these organelles, suggesting that oxidation of dolichols on the double bonds with formation of epoxides does not occur inside NM-containing organelles. The conclusion that dolichols are not synthesized within NM-containing organelles is further supported by the absence of two key enzymes required for dolichol synthesis, i.e., dehydrodolichyl diphosphate synthase complex subunit DHDDS (DHDDS) and polyprenol reductase (SRD5A3) which were not detected by LC-MS, WB, or IEM (Supplementary Fig. 5, 6).

The presence of dolichols, dolichoic acids, and other lipids we describe in the NM-containing organelle has never been reported in previous studies. In the past, dolichols, dolichoic acids, and other lipids were reported only in isolated NM pigment, a different situation, as they can be adsorbed into NM during isolation and originate from cytosol and other organelles that are broken during the isolation process.^28,42,43^ Thus, in the present study we provide the demonstration of the presence of these lipids in the lipid bodies of intact NM-containing organelles.

## Discussion

### Integrated methodology for NM samples preparations and the use of different analytical methods

The study of protein and lipid pathways of NM-containing organelles requires highly purified and well preserved organelles. This is challenging because during their preparation, membrane and soluble proteins can be lost and contamination of organelles by proteins or lipids arising from other cellular compartments may occur. Another factor is that human brain tissues used for preparation of organelles are *post mortem* and thus affected by degradation.^44^ The preparation of organelles can itself provide a source of changes in the distribution of proteins and lipids, as discussed in the first paragraph of Results.

A previous report analyzed by LC-MS/MS the NM-containing organelles isolated from SN tissues, but that isolation procedure was different than that used here and started from frozen tissues.^23^ It is well known that freezing and thawing of *post mortem* tissues can break the membranes, with leakage of proteins that can diffuse among organelles as a possible source of contamination. A later study proposed a new centrifugation method for the combined isolation and enrichment of NM granules (i.e., NM-containing organelles) and synaptosomes from human SN for proteomic analysis, but again this method processed frozen tissues.^45^ More recently, the same group performed a proteomic analysis on NM-containing organelles obtained by laser capture microdissection from human SN frozen slices.^24^ The clear advantage of this new methodology is in isolating NM-containing samples from very low quantities of tissues, but the laser capture microdissection lacks sufficient resolution to discern among different subcellular components that are clearly present among NM-containing organelles of the tissue (Fig. 1a–c and see previous findings)^1,3,9,10^ and therefore can represent a source of contamination. With such a procedure, the collected samples inevitably would contain different types of debris deriving from other cellular components.

Additionally, the LC-MS/MS employed here for proteomic analysis provides a multidimensional protein identification technology method, which is an excellent gel-free approach and provides improved selectivity and resolution of peptide separation, with an increased number of identified proteins and better quantitative determinations in complex mixtures.^46^ In order to improve the identification of protein and lipid pathways, and to distinguish proteins and lipids related to different components of the NM-containing organelle, we analyzed three different NM preparations (ORG, TIS-NM, and ORG-NM): the NM-containing organelles isolated from SN, NM pigment isolated from SN tissues, and NM pigment isolated from NM-containing organelles. The localization of some proteins in NM-containing organelles and other cellular organelles was also confirmed by IEM in intact SN tissue slices.

Notably, in the present study, fresh (rather than frozen and thawed) SN tissue was examined and the isolation procedure enabled us to obtain highly purified NM-containing organelles with well preserved membranes and lipid bodies, as demonstrated in electron micrographs (Fig. 1c). The only contaminant rarely observed in ORG preparations were a few red blood cells, and so their associated previously identified proteins^47,48^ were subtracted from the proteomic data sets of ORG samples (Methods).

It is noteworthy that the larger mass of our samples in any of the three types of preparations contained the same 35 proteins (corresponding to ∼80 % of the overall SpC of representative proteins detected in all samples, as shown in Fig. 3), thus demonstrating the high reproducibility and pertinence of the detected proteins in the NM-containing organelle.

Euler diagrams (Supplementary Fig. 8) show the comparison of our proteomic data with those previously reported by Tribl et al. and Plum et al.^23,24^ Considering all the proteins here identified (Supplementary Fig. 8a), there are 50 proteins found also within the 72 proteins (∼69 %) identified by Tribl and colleagues.^23^ Among these overlapping proteins, we observed that 36, 32, and 34 proteins belong to ORG, TIS-NM, and ORG-NM respectively, representing ∼6 %, ∼8 %, and ∼15 % of all proteins detected in each of the three types of samples (Supplementary Data 1; Table 1). The observation that NM-containing organelles analyzed in the mentioned study^23^ had the highest similarity with our NM pigment isolated from organelles (ORG-NM) suggests that samples in that study featured broken membranes and contained mainly proteins strictly bound to NM pigment. In the present study, we isolated the NM pigment from its organelle by intentionally breaking membranes with a freeze/thaw procedure, while Tribl and colleagues isolated the NM-containing organelles from SN frozen tissues.

In parallel, the comparison between the list of all the proteins we identified and the list of 1000 proteins reported by Plum and colleagues^24^ shows that 188 proteins (∼19 % of proteins identified by Plum’s group) are shared by the two studies (Supplementary Fig. 8a). A more detailed analysis of the overlapping proteins shows that 102, 99, and 71 proteins belong to ORG, TIS-NM, and ORG-NM respectively, representing the ∼18 %, ∼24 %, and ∼32 % of all proteins detected in each of the three types of samples (Supplementary Data 1; Table 1). Also in this case, the ORG-NM sample shows the highest similarity with the samples analyzed by Plum et al. (Supplementary Data 1).

Among new proteins related to NM-containing organelles detected by LC-MS, here we report some lysosomal proteins that were not found in the two previous studies: e.g., iduronate 2-sulfatase, carboxypeptidase Q, lysosomal acid phosphatase, lysosomal alpha-mannosidase, and ribonuclease T2. In addition, we reported many other proteins of noticeable interest among those never identified before as associated with NM-containing organelles: examples include HLA, GPNMB, transmembrane protein 106B, F-box only protein 11, protein phosphatase 1 regulatory subunit 1B, intraflagellar transport protein 81 homolog, etc. (Supplementary Data 1). Some of these proteins have been independently confirmed by IEM and/or WB as discussed below.

Finally, it should be noted that there are 43 proteins shared by our study and other two studies,^23,24^ considering all the proteins we detected (Supplementary Fig. 8a; Supplementary Data 1). If we evaluate the most enriched proteins detected in all the studies by using different samples and methodologies, we should overlap our representative proteins with the 166 significantly overrepresented proteins reported by Plum’s group and with those detected by Tribl et al. The result of this evaluation is a small group of 18 proteins (Supplementary Fig. 8b), 16 of which are lysosomal proteins. If we exclude these 16 typical lysosomal proteins, there are two particularly interesting proteins detected in all three studies. These two proteins, both primarily assigned to endoplasmic reticulum, are phospholipase D3 and protein disulfide-isomerase A3, which seem to be closely related to NM-containing organelles and are briefly discussed below.

### The NM-containing organelle is an autophagic lysosome with particular catabolic features

Protein analyses of the three different types of NM-derived samples (ORG, TIS-NM, and ORG-NM) revealed that lysosomal proteins are the major class of proteins in the NM-containing organelle, representing ~60 % of overall representative peptides identified in all samples (Fig. 2; Supplementary Table 1). Comparison of our data with recent lists of defined human lysosomal proteins^38,49,50^ indicates good overlap but also some differences in the distribution of lysosomal enzymatic classes. In particular, the Euler diagrams (Supplementary Fig. 9) and additional table (Supplementary Data 3) show the comparison between our data and the study by Sleat and colleagues,^38^ which is the most detailed human brain proteomic study performed by detecting only the soluble resident lysosomal proteins using mannose 6-phosphate (Man-6-P) as an univocal lysosomal marker. Among all the proteins here identified (Supplementary Fig. 9a), we found 26 of the 48 (∼54 %) confirmed lysosomal soluble proteins of human brain;^38^ if we consider our representative proteins only, we found 22 of 48 (∼46 %) confirmed lysosomal proteins (Supplementary Fig. 9b).

It thus appears that peptidases and a majority of esterases are overrepresented in our samples, while lipases and glycosylases are underrepresented (Supplementary Data 3). In detail, 10 of 14 peptidases (E.C. 3.4.-) belonging to human brain lysosomes^38^ were detected and in large amounts in our samples (with overall 3243 SpC, corresponding to ∼41 % of lysosomal SpC), suggesting again an overrepresented complement of protein degradation pathways in NM-containing organelles (see also first paragraph of Results). The identification of CTSD in its heavy chain (mature form) as abundant in all samples, revealed by LC-MS data and confirmed by WB and high IEM gold signals, is notable considering its role in limiting lysosomal storage diseases^51^ and in inhibiting SNCA aggregation.^52^ Similarly, 6 of 11 lysosomal esterases (E.C. 3.1.-) identified in human brain lysosomes^38^ were detected by overall 2523 SpC, corresponding to ∼32 % of lysosomal SpC (Supplementary Data 3). Among these proteins, sialate *O*-acetylesterase, a key enzyme involved in sialic acid catabolism, was the lysosomal esterase we detected by the highest number of peptides (i.e., overall 2043 SpC).

In contrast, typical lysosomal human brain enzymes mainly involved in lipids, phospholipids, and sphingolipids catabolism, including lysosomal acid lipase, group XV phospholipase A2, and sphingomyelin phosphodiesterase were not detected. We observed only elevated quantities of PLBD2 and phospholipase D3, two poorly characterized proteins of unknown functions, that were identified both as potential proteins of human brain lysosomes^38^ and in previous studies on NM-containing organelles.^23,24^ PLBD2, also confirmed and localized by IEM signals, is a new putative lipase^37,38^ with uncertain enzymatic activity, with the exception of a homolog protein in amoeba that cleaves acyl chains of some phospholipids (phosphatidylinositol, phosphatidylethanolamine, and phosphatidylcholine).^53^ Due to the accumulation of dolichols in NM-containing organelles, and considering that dolichols may be transported to lysosomes as dolichyl esters and then hydrolyzed by an unknown dolichyl esterase,^54^ we attempted to test by molecular docking if dolichyl esters are possible substrate of PLBD2, but obtained no fit (not shown). Similarly, despite its classification as an esterase, neither a definite enzymatic activity nor specific substrates have been clearly reported for phospholipase D3.^55,56^ Nevertheless, phospholipase D3 is abundantly expressed in brain and neural tissues,^55,56^ is correlated with the modulation of cellular resistance to oxidative stress^57^ and has been recently indicated as a key factor in the pathological processes of Alzheimer’s disease.^58^ The finding of large amounts of both of these two recently discovered enzymes in all samples indicates a need for further investigation into their roles in NM-containing organelles and lysosomes, in particular on metabolism and storage of lipids.

Another group of less represented enzymes are glycosylases: indeed, only 5 of at least 14 enzymes classified as glycosylases and reported by Sleat and colleagues (Supplementary Data 3), principally involved in glycoproteins, glycosaminoglycans and glycosphingolipids degradation pathways in lysosomes,^59,60^ were detected and in very low amounts (overall 49 SpC, <1 % of lysosomal SpC). Concerning the shortage of enzymes related to catabolic pathways of lipids in our samples, an exception is acid ceramidase and its specific prosaposin (which undergoes proteolytic cleavage to form saposins), both involved in the last step of sphingolipids degradation pathway in lysosomes.^60^ Acid ceramidase and prosaposin were found in NM-containing organelles by relative high amount of peptides (overall 225 SpC and 70 SpC, respectively).

Thus, it appears that NM-containing organelles possess a low representation of typical components of phospholipids and sphingolipids degradation pathways. This could indicate that NM-containing organelles lose the ability to conduct specific enzymatic pathways as it accumulates in neurons, and could be related to the particular lipid storage content of NM-containing organelles, consisting mainly of dolichols,^3,42^ for which catabolic pathways are still unclear and unrelated to phospholipid/sphingolipid pathways. On the other hand, our findings are consistent with the presence of undegraded glycerophospholipids and sphingolipids in the lipid extract from the lipid bodies.

### Lysosomal membrane proteins are less represented in NM-containing organelles than conventional lysosomes

Among lysosomal membrane proteins, we detected by different techniques SCARB2, LAMP1, LAMP2, CD63 antigen, type 1 phosphatidylinositol 4,5-bisphosphate 4-phosphatase, and some V-type proton ATPase subunits. In particular, SCARB2 was shown by LC-MS, IEM and WB to be the most abundant lysosomal membrane protein in NM-containing organelles. One function of SCARB2 may be to transport β-glucocerebrosidase into the lysosome,^61^ although as above β-glucocerebrosidase was not identified in this study by LC-MS. SCARB2 also belongs to the scavenger receptor class B family involved in the transport of high density and low density lipoproteins, cholesterol esters, phospholipids and oxidized phospholipids,^62,63^ and if overexpressed, induces endosomes/lysosomes enlargement and cholesterol accumulation in the enlarged compartments.^64^ The abundance of dolichols into the NM-containing organelles could be related to the presence of high amounts of SCARB2 in NM-containing organelles. This protein is present both on the organelle membrane and its lumen, especially in lipid bodies and sometimes in NM pigment, and is apparently accumulated in the NM-containing organelle during aging (see below).

Other lysosomal membrane components were identified to a lesser extent, including LAMP1, LAMP2, CD63 antigen, type 1 phosphatidylinositol 4,5-bisphosphate 4-phosphatase, and some subunits of V-type proton ATPase. Considering the probable partial loss of membranes during isolation procedures and/or due to problems in detecting membrane proteins by LC-MS, IEM and WB analyses were performed, which demonstrated the presence of LAMP1, LAMP2, and ATP6V1B2 subunits, mainly in the luminal portion of the NM-containing organelles. The antibody used for LAMP2 detection by IEM and WB recognizes all three LAMP2 isoforms, and so we cannot distinguish between LAMP2A (the chaperone-mediated autophagy receptor), LAMP2B (probably involved in macroautophagy), and LAMP2C.^65^ Autophagosomes possess LAMP2B and LAMP2C isoforms, which have uncertain functions distinct from chaperone-mediated autophagy.^66^ However, LAMP2 appears to have a low presence with abnormal location in the NM-containing organelle, which could be consistent with the age-related decreased level of this protein, particularly for LAMP2A in lysosomes,^67^ as well as a general decline of autophagic-lysosomal function that occurs in normal aging. These observations suggest that NM-containing organelles are likely derived from macroautophagic organelles (see next paragraph), rather than lysosomes specialized for chaperone-mediated autophagy.

The identification by LC-MS of only low levels of V-type proton ATPase functional subunits, responsible for acidification of lysosomes, may indicate a decreased acidification and diminished lysosomal catabolism. As vacuole fusion requires an electrochemical membrane potential created by the V-type proton ATPase, NM-containing organelles may have a low capacity for fusion with lysosomes or autophagosomes. IEM experiments revealed that ATP6V1B2 subunit is not evident on the membrane of NM-containing organelles (in contrast to lysosomes) but is sparsely located in NM pigment and lipid bodies, confirming a likely functional deficiency of V-type proton ATPase in the NM-containing organelles.

### The NM-containing organelle originates from macroautophagy and accumulates MAP1LC3B

Little is known about the origin of the NM-containing organelle. Experiments in cultured neurons showed that an excess of cytosolic DA induces NM synthesis and NM-containing organelles formation, and the induced pigment was chemically identical to human NM as assessed by electron paramagnetic resonance.^2,10^ In addition, the identification of a double membrane around these induced organelles of NM and surrounding NM-containing organelles of the human SN, as previously reported^2,10^ and here confirmed (Fig. 1c), together with presence of many lysosomal hydrolases, suggests that the NM-containing organelle is a pigmented autophagic vacuole.^2^

We have shown in previous studies electron microscopy images of NM-containing organelles of human brain that have a clearly different ultrastructural appearance than lipofuscin and lysosomes.^2,3,9,10^ Another paper described the species-specific ultrastructure of neuronal lipofuscin by electron microscopy, thus showing morphological features of lipofuscins that are quite different from that of NM-containing organelles.^68^

**Fig. 8:**
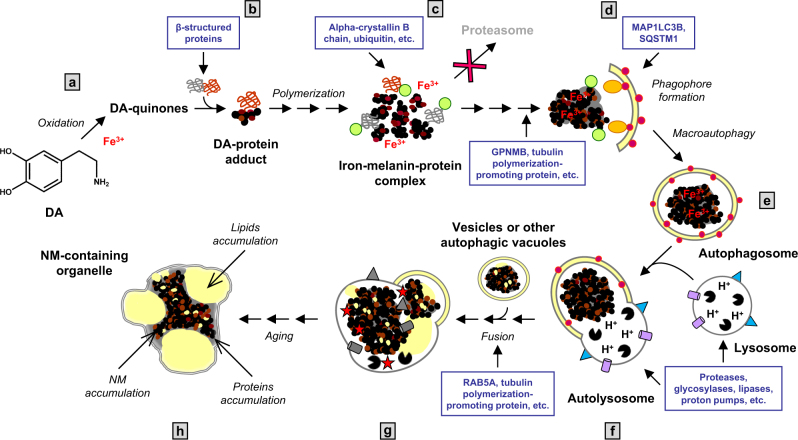
Hypothesized scheme summarizing NM-containing organelle formation in human SN. **a**, **b** In the cytosol of SN neurons, DA can be oxidized to semiquinones and quinones via iron catalysis, and these highly reactive compounds can react with aggregated and β-structured proteins that accumulate in the cytosol. **c** The oxidative polymerization of quinones initiates with the formation of the melanin-protein complex which can also bind high levels of metals, especially iron. During this step drugs and toxicants can also bind to the melanin-protein complex.^18^ Proteins damaged by misfolding and DA-adducts formation may be recognized and bound by ubiquitins (green) and alpha-crystallin B chain, in the attempt to degrade damaged and/or misfolded proteins in the proteasome pathway. It may be that ubiquitinated-NM-derived products are too large and damaged to be degraded by the proteasome system.^91^ **d**, **e** The resulting undegradable material accumulates in the cytosol. GPNMB and tubulin polymerization-promoting protein could be engaged at this step, since these proteins are involved in the formation of aggresome-like structure and degradation of cellular debris.^105,110^ The accumulated undegradable material is then taken up into autophagic vacuoles by the phagophore, an isolation double membrane which engulfs bulk material for macroautophagy. This is confirmed by the presence of some typical macroautophagic markers^94^ such as MAP1LC3B (red) and SQSTM1 (orange), and by the presence of a double membrane surrounding the NM-containing organelle as displayed by electron microscopy (Fig. 1c)**. f** These autophagic vacuoles fuse with lysosomes, shown in the scheme with numerous enzymes (black), different membrane proteins (blue) and the proton pumps (violet), to become autolysosomes containing the enzymes, proteins and lipids of lysosomes. After fusion with lysosomes, the undegraded and NM-derived material contained in the autophagic vacuoles can interact with other lipids and other proteins carried by lysosomes. A decreased lysosomal enzyme efficiency and reduced fusion capacity could occur also as a consequence of aging, oxidative stress and NM accumulation. **g** These organelles can fuse with other vesicles or with other autophagic vacuoles containing NM precursors or with old NM-containing organelles, etc. This fusion could lead to the accumulation in the lumen of the organelle of membranous portions which would otherwise be degraded, while dolichols in particular are not chemically decomposed and accumulate with other undegraded lipids leading to the formation of lipid bodies. Dolichols (or dolichyl esters) may be transported into the organelle by vesicle transport and membrane fusion. Fusion of organelles could be mediated by RAB5A, tubulin polymerization-promoting protein, microtubule-associated protein tau and other related proteins. This aged organelle, due to its particular content of undegradable NM pigment together with damaged and oxidized proteins, lipids and metals, is a reservoir of buffered toxins (red stars). Under conditions of cellular damage these toxins together with NM pigment could be released and induce neuroinflammation and neurodegeneration.^19^ **h** The final NM-containing organelle is the result of a complex and continuous process occurring during aging, that leads to the accumulation of undegradable material in specialized pigmented “autophagic lysosomes”. Figure modified from previously published papers, by permission of Springer^13^ and by permission of Elsevier^14^

Here we report the presence of the macroautophagy marker MAP1LC3B inside NM-containing organelles, confirming its autophagic nature. Using IEM on SN tissues, we found MAP1LC3B localized around lipid bodies, lining membranes and remarkably on NM pigment within organelles. This evidence confirms that NM-containing organelles may derive by formation of MAP1LC3B-positive autophagosomes that engulf forming NM and related proteins and lipids components present in the cytosol, and successively fuse with lysosomes, forming NM-containing autolysosomes that become NM-containing organelles (Fig. 8).

The level of MAP1LC3B-II is thought to be a reliable indicator of autophagosome formation.^69^ However, in our ORG samples, the MAP1LC3B-II form was undetectable by WB compared to MAP1LC3B-I, either because it was delivered at low levels or because it was normally degraded by lysosomal enzymes. It is likely that MAP1LC3B-II form could be degraded by the lysosomal hydrolases we found, as normally occurs after the fusion of our special autophagosomes with lysosomes (Fig. 8), and therefore this transient supply of lysosomal enzymes would degrade some substrates. Indeed, after autophagosome fusion with lysosomes to form autolysosomes, intra-autolysosomal MAP1LC3B-II is normally degraded by lysosomal hydrolases and is difficult to detect.^70^

### The NM-containing organelle is an aged and impaired lysosomal-related organelle that accumulates proteins, indigestible NM, and dolichols

In addition to acid ceramidase, other proteins (as previously described) including tripeptidyl-peptidase 1 and APOD were present in high amounts in the samples and have been also reported as major components of lipofuscin-like lysosomal inclusion bodies.^71^ APOD is involved in binding and transport of lipids,^72^ in their protection from oxidation and consequent oxidative stress.^73^ APOD is consistently upregulated and highly expressed during normal aging,^74^ in overall SN tissue of PD patients^75^ and other neurodegenerative diseases,^72^ where oxidative stress and lipid abnormalities are implicated. As mentioned, SCARB2 accumulates in the NM-containing organelle as also reported for lysosomal inclusion bodies.^71^ While SCARB2 is a protein of the lysosomal membrane, it was also found on the luminal side of the NM-containing organelle particularly in lipid bodies and sometimes in NM pigment. Although some of these proteins may be enriched in the NM-containing organelle due to their particular role or because they are normally overexpressed during aging, these proteins may be also accumulated due to impaired degradation in NM-containing organelles.

We note that ATP5G1/2/3, that we found in NM-containing organelles, is the primary marker of broad range of neuronal ceroid lipofuscinoses (i.e., CLN2, -3, -4, -5, -6, -7, -8, -9, CLCN7).^36^ ATP5G1/2/3 accumulates in autophagic vacuoles and lysosomes of neurons where autophagy or some lysosomal enzymes are blocked, as in lysosomal storage disorders.^76,77^ Intriguingly, this protein also accumulates inside autophagic vacuoles in normal aged mice,^76^ consistent with a decline of autophagic-lysosomal function during normal aging. Similar to the age-dependent accumulation of dolichols in brains of the elderly,^78,79^ which was greatly increased in patients with neuronal ceroid lipofuscinosis,^80,81^ ATP5G1/2/3 accumulates in lipofuscin-like lipopigments inside normal neurons during aging, a process amplified in neuronal ceroid lipofuscinosis and other lysosomal disorders.^82^ Interestingly, these previously reported observations are consistent with the LC-MS detection of this protein specifically in TIS-NM samples, which can be associated to the early stages of NM-containing organelle formation. Additionally, saposins, which were detected in our samples in the form of prosaposin, have been identified as the main storage material (particularly saposins A and D) in two types of neuronal ceroid lipofuscinosis, CLN1 and CLN10.^36^

Other proteins accumulated inside NM-containing organelles are cerebellin-1 and cerebellin-2, which have unknown functions. Cerebellin-1 (which we found in all analyzed samples) is preferentially expressed in cerebellar synapses, where it is required for synapse integrity and plasticity, but is also present at variable concentrations elsewhere in the brain, and a study reports its presence in the endo-lysosomal compartments of neurons.^83^ Cerebellin-1 is more highly expressed in SN (A9) neurons than ventral tegmental area (A10) neurons of mice,^84^ suggesting a mechanism for its accumulation similar to that of NM in dopaminergic neurons of SN, and is also highly expressed in mucopolysaccharidosis type IIIB mouse brains.^85^

Previous studies have suggested the storage features of the NM-containing organelle: (i) indigestible NM pigment increases in concentration in neurons from very early life over the entire life span;^3–6^ (ii) NM pigment binds high quantities of dolichols;^3,9,42,43^ and (iii) NM pigment accumulates large amount of endogenous and environmental metals.^3,15^ We now report the identification of storage material in these aged organelles, suggesting that mechanisms for degrading protein and lipid components are impaired, despite the presence of several enzymes inside NM-containing organelles. This evidence, together with low levels of some catabolic lysosomal enzymes and shortage of lysosomal membrane proteins (as discussed in the previous sections on lysosomal proteins), including V-type proton pump ATPases involved in acidification, suggests that the NM-containing organelle is an aged impaired lysosomal-related organelle unable to completely digest its content.

This organelle, due to its particular content of undegradable NM pigment, proteins, lipids and metals (see below), could be also a source of oxidative stress (Fig. 8). Therefore, the presence of proteins involved in protection against oxidative stress are notable: i.e., phospholipid hydroperoxide glutathione peroxidase, protein DJ-1, superoxide dismutase [Cu–Zn], and APOD. Here, we briefly highlight two examples. Phospholipid hydroperoxide glutathione peroxidase, which contributes to redox balance in cells, protects lipid membranes from oxidation and was previously observed to co-localize with NM pigment of dopaminergic SN neurons. Indeed, this hydroperoxidase was significantly reduced in overall parkinsonian SN compared to controls, but was increased in the surviving SN neurons.^86^ Protein DJ-1 has been identified as an atypical peroxidase that scavenges hydrogen peroxide derived toxicity^87^ and mutations in its gene are associated with early-onset PD.^88^

We investigated FTH1 and FTL, proteins involved in iron homeostasis/storage, in NM-containing organelles. Using immunohistochemistry, we previously reported that FTH1 and FTL content in NM-containing neurons of SN is much lower than that of neurons not containing NM or glia. Oligodendrocytes showed the highest positive reactions for both FTL and FTH1, while in neurons enriched with NM-containing organelles the staining for FTH1 and FTL was generally undetectable with this technique.^5,6^ Due to technical limitations of peroxidase immunohistochemistry, low quantities of these iron storage proteins could not be revealed. By using techniques with high sensitivity (LC-MS and IEM), we now confirm the presence of FTL previously reported^89^ and, to a lesser extent, of FTH1 in NM-containing organelles of the SN. In conclusion there are low levels of ferritins inside NM-containing organelles, while the abundant NM pigment is the major iron storage complex of pigmented neurons.^3,5,6,14^

The presence of HLA in NM-containing organelles and its accumulation in NM pigment may have important consequences for preferential vulnerability of catecholaminergic pigmented neurons in SN and locus coeruleus of PD patients. HLA expression is higher in SN and locus coeruleus pigmented neurons than other brain neurons, and SN dopaminergic neurons in culture can express HLA that can bind peptides from endogenous or exogenous proteins and present them on neuronal membrane leading to targeting by CD8^+^ lymphocytes and death.^20,90^

### Proteins involved in aggregation, degradation pathways, and potential precursors of NM synthesis

The presence of abundant UBC and UBA52 in the NM pigment inside the NM-containing organelles, as demonstrated by LC-MS and IEM, suggests that ubiquitinated proteins likely participate in early steps of NM synthesis in the cytosol. In addition, WB data demonstrate that high levels of ubiquitinated proteins with high molecular weight are detected in the NM-containing organelle. Ubiquitination can direct proteins, particularly when partially unfolded or damaged, to either proteasome or lysosome; if proteasome is unable to degrade all ubiquitinated proteins, macroautophagy could provide an important compensatory mechanism.^91–93^

Ubiquitination probably occurs during NM formation attempting to degrade proteins damaged by DA-modification. Proteins may be modified by DA-quinones, as we have previously detected by chemical degradation of NM pigment isolated from SN (TIS-NM) high amounts of cysteinyl adducts with DA and with 3,4-dihydroxyphenylalanine (DOPA), in addition to DA and DOPA.^3^ This suggests that quinones of DA and DOPA are trapped by cysteine residues of proteins (although histidine residues can similarly react with quinones), producing DA- and DOPA-modified proteins during the early steps of NM biosynthesis. It may be that ubiquitinated-, NM-derived products are too large to be degraded by the proteasome and therefore are removed by macroautophagy and finally stored in the NM-containing organelle (Fig. 8). We further detected SQSTM1, an important partner of MAP1LC3B, required for the degradation of ubiquitinated aggregates by autophagy.^94^ Interestingly, SQSTM1 accumulates in ubiquitin-rich inclusion bodies in neurodegenerative protein aggregation diseases.^95,96^ The formation of protein inclusion bodies enriched in SQSTM1 and ubiquitins is a typical response to stress conditions including amino acid starvation, oxidative stress, and inhibition of lysosomes and autophagy.^94,97,98^

Alpha-crystallin B chain and heat shock protein HSP 90-alpha were found in our samples and could play a role similar to that of SQSTM1 and ubiquitins during NM-containing organelle formation. Heat shock protein HSP 90-alpha is a chaperone that promotes protein folding and might rescue damaged proteins,^99^ and has been detected also in melanosomes.^100^ Alpha-crystallin B chain is a small heat-shock protein that can function as a molecular chaperone and prevents fibrillization of proteins, particularly SNCA:^31,101^ it is sometimes present with ubiquitinated proteins and SNCA in Lewy bodies,^102^ and recently was found markedly upregulated in the SN of PD patients.^103^ The finding of this protein in both the ORG and ORG-NM, but not the TIS-NM sample, suggests that the protein might not be strictly bound to NM pigment and is present as a component of the protein matrix of the NM-containing organelle.

Protein disulfide-isomerase A3, a protein found only in TIS-NM sample and then strictly bound to NM pigment, was also reported by previous proteomic studies as non-lysosomal protein of NM-containing organelles.^23,24^ This enzyme catalyzes disulfide bond formation, reduction, or isomerization and belongs to a family responsible for quality-control system to ensure the correct folding of proteins. Interestingly, a member of this family of proteins was shown to co-localize with SNCA in brainstem and cortical Lewy bodies of subjects with neurodegenerative diseases.^104^

Tubulin polymerization-promoting protein, a protein involved in the maintenance of microtubule network stability,^105,106^ plays also a role in SNCA aggregation and co-localizes with aggregated SNCA in Lewy bodies inclusions in a group of α-synucleinopathies.^30^ We found tubulin polymerization-promoting protein in ORG and ORG-NM samples, suggesting its possible involvement in NM synthesis and NM-containing organelle formation due to its reported role in initiating the formation of cellular aggresome-like structures and inclusion bodies.^105^

The presence of GPNMB in all isolated samples, as also confirmed by WB and IEM, is remarkable. The melanosomal protein GPNMB (refs. ^107,108^^)^ is found in melanosomes of MNT-1 cells by proteomics analysis,^100^ participates in melanogenesis,^109^ and in control of macroautophagy and bulk degradation in the cytosol, as a recruiter for MAP1LC3B.^110^ GPNMB could play a role in formation of NM autophagic vacuoles and fusion to produce the final NM-containing organelle. Notably, its corresponding gene is a new PD risk loci reported in a wide association meta-analysis.^111^ This suggests that mutations of this gene could encode a modified GPNMB protein unable to participate in the macroautophagic process producing the NM-containing organelle, leaving the neuron exposed to toxic species. A recent gene expression study on 6-hydroxydopamine animal models of PD revealed that GPNMB (as well as other genes belonging to regeneration-associated genes) was highly upregulated in SN early after the lesion.^112^ This upregulation could be a response associated with axodegenerative process of SN neurons after lesions, and could also exhibit axoprotective or regenerative properties.^112^

### The lipid bodies of NM-containing organelles contain mainly dolichols

The lipid bodies in NM-containing organelles contain mostly dolichols and dolichoic acids, although we did not find proteins for dolichol synthesis or transport, including two important enzymes for the last steps of synthesis of dolichols (DHDDS and SRD5A3).^113^ No dolichol-specific transporter has been yet characterized, and only a single study to our knowledge reported that dolichols intermediates in the human blood are normally transported by low-density lipoproteins and may be involved in their accumulation in lysosomes during normal aging and lysosomal diseases.^114^ However, we could not detect the receptor for low-density lipoproteins as representative in our samples. Dolichols may exist as free forms with free terminal hydroxyl group, or in phosphorylated forms (dolichol phosphate is required for glycoprotein biosynthesis), or esterified with fatty acid, which is the dominant form of dolichols in animal tissues.^113,115^ It is possible that esters of dolichols with fatty acids (dolichols are usually transported to the lysosomes in the esterified form)^54^ are transferred into NM-containing organelles, where they could be slowly hydrolyzed to free dolichols by the abundant hydrolase activities (esterases) we detected. The intracellular accumulation of dolichyl esters may occur because a majority of lipids are degraded by cytosolic, lysosomal, and mitochondrial enzymes, while dolichols are poorly degraded by unknown pathways and unidentified specific catabolic enzymes.^116,117^ It may be that dolichols (or dolichyl ester) can be transported into the NM-containing organelle by vesicle transport and membrane fusion from other organelles (other NM-containing organelles, lysosomes, etc.). Indeed, the largest lipid bodies present into the NM-containing organelles are mainly located on the external portion of organelle and are membrane bound (Fig. 1a–c), although we cannot distinguish between a normal bilayer or a single layer; the smallest lipid bodies are observed both embedded into NM pigment and in the external portion of the organelle.

Although the precise role of dolichols is not established, they may be important for membrane trafficking,^118^ and stimulating fusion of lipid vesicles.^119^ It is possible that dolichol-containing membranes continuously fuse during NM-containing organelle formation and maturation (NM-containing autophagosomes with new lysosomes, with old NM-containing organelles, etc.), and consequently, dolichols may accumulate in NM-containing organelles that are unable to efficiently degrade them (Fig. 8). Thus, dolichols in the organelle could derive from incompletely degraded membranes of other organelles.

The phospholipids and sphingolipids we found in NM-containing organelles likely derive from the organelle’s own membranes and incomplete degradation of membranes of autophagic vacuoles that fuse with NM-containing organelles, as well as by vesicle transport into NM-containing organelles.

Sulfatides are synthesized and accumulated predominantly in oligodendrocytes and are a major component in myelin sheaths, although low amounts of sulfatides have been detected in neurons and astrocytes.^120^ The sulfatides accumulated in NM-containing organelles (at very low level in ORG samples compared to purified TIS-NM, see Results) could derive from disruption of oligodendrocytes, dopaminergic and non-dopaminergic axons and enter the NM-containing organelles through endocytosis, vesicle transport and fusion. NM-containing organelles are also characterized by the presence of some typical neuronal sphingolipids, for example gangliosides (as revealed by LC-MS), which likely do not undergo complete degradation due to the loss of activity of glycosyl hydrolases involved in their catabolism, consistent with the notion that the NM-containing organelles are a form of aged and impaired lysosomal-related organelle.

### NM synthesis begins in cytosol and intermediate products are transported into organelles

We did not find significant amounts of typical enzymes and proteins involved in melanogenesis (i.e., tyrosinase, dopachrome tautomerase, melanocyte protein PMEL17, etc.),^100,121,122^ with the sole exception of the melanosomal protein GPNMB, a glycoprotein with high homology to the structural melanocyte protein PMEL17.^108,122^ In addition to the role suggested above for GPNMB in autophagy to form the NM-containing organelle, this protein could produce amyloid fibrils assembling into sheets on which DA-quinones would start NM synthesis (Fig. 8). Indeed, it was shown that in the absence of glycosylation, the NTR-PKD domain region of GPNMB retains the intrinsic capacity to form amyloid in cell cultures.^123^

Likewise, we did not find DA transporters, with the sole exception of the synaptic vesicular amine transporter which was detected at very low levels only in ORG-NM sample and categorized as non-representative. However, it was shown in the past that human DA neurons of SN with high levels of NM pigment have low levels of synaptic vesicular amine transporter expression and *vice versa*.^12^ Similarly, the overexpression of synaptic vesicular amine transporter in neuronal cell cultures, treated with l-DOPA which is rapidly converted to DA, inhibited the synthesis of NM by lowering the cytosolic concentration of DA.^10^ It is noteworthy that the protein phosphatase 1 regulatory subunit 1B, that we found in all samples, is involved in l-DOPA induced dyskinesia in PD. The l-DOPA increases cytosolic DA and protein phosphatase 1 regulatory subunit 1B phosphorylation.^124,125^ This protein could be involved in the first steps of NM synthesis by reacting with high cytosolic DA and related catecholamines or their derived products (semiquinones and quinones), due to the presence of cysteines and histidines in its structure. Another protein that may react with quinones during the early steps of NM synthesis could be the cysteine-rich protein 2, which was detected in ORG-NM samples. More broadly, candidate proteins that could be incorporated into NM pigment during the early phases of biosynthesis are those containing a high number of cysteine and histidine groups, which readily react with quinones.^126^

It appears that enzymatic (if any) oxidation of DA to form melanic oligomers occurs in the cytosol, likely via iron catalysis or by some of the oxidative enzymes (Fig. 8). Evidence for enzymatic control of the oxidative process leading to NM pigment formation has been discussed but has not been demonstrated directly.^13,14,127,128^ Interestingly, we found two oxidoreductase enzymes in TIS-NM sample by LC-MS. The first is the 3 beta-hydroxysteroid dehydrogenase type 7 (found exclusively in TIS-NM sample), which is normally localized in the endoplasmic reticulum. The other enzyme is the superoxide dismutase [Cu–Zn], normally found in the cytoplasm, which was found in TIS-NM with SpC higher than that of the previously mentioned enzyme, and was also found with slightly higher SpC as fragment in the ORG sample. This suggests that superoxide dismutase [Cu–Zn] could be an additional candidate enzyme involved in the oxidation of DA and related catecholamines during the early phases of NM synthesis. In addition, the existence of an auto-oxidation process of DA and related catecholamines must be also considered in the biosynthesis of NM pigment, a process that to date is only partially understood. The presence of iron(III) promotes the oxidation of DA into highly reactive quinones that can form NM pigment.^14^ Indeed, NM synthesis may be driven by an excess of cytosolic catecholamines not accumulated in synaptic vesicles, as suggested previously.^10^ Moreover, the oxidation of DA to semiquinones and quinones can generate aminochrome and 5,6-indolequinone, which can induce toxicity if these species are not taken into NM synthesis. These reactive compounds are reported to cause dysfunction in mitochondria and protein degradation, and to promote aggregation of SNCA to toxic protofibrils and produce oxidative damage.^129^

The melanic component derived from the oxidative polymerization of DA and/or other related catecholamines likely binds to β-structured proteins, since X-ray scattering studies^3^ performed on isolated NM pigment showed a diffraction pattern of about 4.7 Å, typical of cross-β sheet structured protein aggregates.^130^ Since GPNMB and SNCA (both capable of forming insoluble fibrils with cross β-structure, and WB on ORG samples indicate the presence of complex aggregates) were found in different parts of the NM-containing organelle, including NM itself, we propose that NM formation may start with the accumulation of β-structured protein aggregates in the cytosol, resulting in the formation of peptide/protein “seeds”, either protofibrils or even fibrils, which would react with excess of DA and/or quinones of DA metabolites,^131^ followed by polymerization to form melanin-protein conjugates. The protein-DA modifications and subsequent polymerization was recently demonstrated in the synthesis of NM models.^126,132^ The oxidation of DA and the resulting complex between melanin and many β-structured proteins, which could include fibrillar forms of SNCA, GPNMB, and other proteins with cross β-structure,^133–135^ is promoted by reactive iron which is abundant in cytosol of SN neurons.^5,14^ Iron(III) could accumulate within the conjugates during this process. Indeed, NM pigment in the organelle binds iron(III) in two distinct iron-binding sites with different affinity.^3,5,14^ This ternary complex of iron-melanin-protein formed in the cytosol can be accumulated into autophagic vacuoles and transported to the lysosome. Here, the proteases would cleave most protein chains, generating a complex with higher ratio of melanin:protein in the final NM pigment.^3,9^ This lower polarity melanin:protein complex will more easily react with dolichols, and to a lesser extent with other lipids, released by lipid bodies present in the organelle to form the insoluble NM pigment which is then continuously accumulated inside membrane-bound organelles. This reaction of the complex iron-melanin-protein with dolichols likely occurs through iron mediated radical oxidation at the carbon atom adjacent to the double bond in the isoprenic unit of dolichols.

We found that dolichols are covalently bound to the melanic component within the NM structure.^3,9,42^ The isolated NM pigment is quite insoluble both in water and organic solvents, although NM pigment contains a relatively low molecular weight component that is slightly soluble in dimethyl sulfoxide with essentially the same composition of the insoluble component.^9^ The insoluble portion seems to contain polymers of larger size, more dolichols and less saturated lipids than its dimethyl sulfoxide-soluble counterpart. The soluble portion of NM pigment likely consists of oligomeric precursors of polymeric NM that have molecular weights between 1.4 and 52 kDa.^9^ The presence of large amounts of oligomeric and polymeric structures further confirms that NM synthesis and accumulation are continuous processes occurring in neuronal organelles (Fig. 8).

## Conclusions

The comparison and integration of data from different methods of preparation and analysis provide a highly reliable and comprehensive description of protein and lipid pathways of NM-containing organelle of the human SN. We found that the NM-containing organelle possesses particular membrane and soluble proteins typical of lysosomes, while other characteristic lysosomal proteins are missing or scarcely expressed. Typical lysosomal enzymes abundant in NM-containing organelles are peptidases, sulfatases and esterases. However, the organelle has lower levels of lipases and glycosylases than typical lysosomes, and so exhibits limited degradation pathways for some molecules. The reduced lysosomal activity and fusion capacity could be a consequence of inadequate localization of some lysosomal membrane proteins, including V-type proton ATPase, which normally acidifies lysosomes.

The LAMP2 is at low levels and inadequately located in NM-containing organelles, suggesting that lysosomes specialized for chaperone-mediated autophagy do not form NM-containing organelles. In contrast, the presence of double membrane surrounding many of the pigmented organelles and the finding of proteins such as MAP1LC3B and SQSTM1 demonstrate the macroautophagic nature of NM-containing organelles, which derive from autophagosomes that engulf NM precursors, lipids and proteins from cytosol.

In lipid bodies of NM-containing organelles, the major components accumulated are dolichols and dolichoic acids, likely transported by SCARB2 and APOD. The high accumulation of dolichols in NM-containing organelles may also be mediated by membrane fusion from other organelles and vesicle transport of dolichyl esters, and their subsequent hydrolysis to dolichols.

The NM-containing organelle accumulates undegradable NM pigment, dolichols, lipids, proteins, and metals over the entire lifespan. Several proteins have been detected in spite of the presence of different proteases and other degradative enzymes, as degradation processes including those dependent on organelle acidification are inhibited or inefficient. Alpha-crystallin B chain and heat shock protein HSP 90-alpha were found as well as tubulin polymerization-promoting protein, GPNMB, ubiquitins (along with several other proteins), likely consistent with their involvement in macroautophagy and bulk degradation in the process of NM synthesis.

Among proteins accumulated inside NM-containing organelles, a striking presence is that of HLA, that could increase the vulnerability of NM-containing neurons, since this protein can present antigens on cell membranes so that neurons would be targeted by T-lymphocytes.

We hypothesize that the NM synthesis starts in the cytosol with accumulation of aggregated and β-structured proteins, which may include SNCA, GPNMB, and other proteins, that bind oxidized DA or DA-derived compounds to produce adducts which undergo further oxidation and polymerization to form the melanic component of NM pigment. The protein-melanin conjugate thus formed traps iron and other metals, and is then accumulated into autophagic vacuoles and carried to lysosomes. Here, the proteases likely cleave most of protein/peptide chains of the protein-melanin conjugate which react with dolichols to form the final NM.

## Methods

### Antibodies

For WB and IEM experiments, the following primary antibodies against different proteins were used: APOD, mouse monoclonal (Abcam, Cambridge, UK); ATP5G1, mouse monoclonal against 18–137 sequence (Abcam), and this antibody recognizes mature chains of ATP5G1, ATP5G2 and ATP5G3, which are identical in their sequence; ATP6V1B2, mouse monoclonal (Santa Cruz Biotechnologies Inc., Santa Cruz, CA, USA); CTSD, goat polyclonal against the C-term (Santa Cruz Biotechnologies); DHDDS, rabbit polyclonal (Atlas Antibodies AB, Stockholm, Sweden); FTH1, goat polyclonal (Abcam); FTL, rabbit polyclonal (Abcam); GPNMB, goat polyclonal against 23–486 sequence (R&D Systems, Minneapolis, MN, USA); HLA, mouse monoclonal (Santa Cruz Biotechnologies); LAMP1, mouse monoclonal (Developmental Studies Hybridoma Bank, Iowa City, IA, USA); LAMP2, mouse monoclonal recognizing all LAMP2 isoforms (Developmental Studies Hybridoma Bank); MAP1LC3B, rabbit polyclonal (Cell Signaling Technology, Danvers, MA, USA) and rabbit polyclonal (Abgent, Inc., San Diego, CA, USA); PLBD2, rabbit polyclonal against 448–565 sequence at C-term (Atlas Antibodies AB); RAB5A, rabbit polyclonal (Abgent, Inc.); SCARB2, mouse monoclonal (Santa Cruz Biotechnologies); SNCA, rabbit polyclonal (EMD Millipore, Temecula, CA, USA); SQSTM1, mouse monoclonal (Abcam); SRD5A3 rabbit polyclonal (Atlas Antibodies AB); UBA52, rabbit polyclonal against ubiquitin (DakoCytomation, Glostrup, Denmark). We have indicated detailed immunogenic sequence only for proteins that undergo relevant molecule processing.

### Brain tissues

This study was approved by the Institutional Review Board of the Institute of Biomedical Technologies—National Research Council of Italy (Segrate, Milan, Italy) and was carried out in agreement with the Policy of National Research Council of Italy. Written informed consents for using brain samples for research purposes were obtained from closest relatives and are stored at the Section of Legal Medicine and Insurances, Department of Biomedical Sciences for Health, University of Milan, Milan, Italy. Final approval was given by the pathologist performing the autopsy. All tissues samples were analyzed anonymously.

In this work, SN samples were obtained during autopsies of male and female subjects who died at different ages without evidence of neuropsychiatric and neurodegenerative disorders. The healthy subjects included in this study at pathological examination did not show any macroscopic alteration of neurological and vascular type. Histological examination (on formalin-fixed and paraffin-embedded tissue sections) revealed no Lewy bodies and other pathological markers.

All samples analyzed and included in this study were obtained from healthy elderly subjects, and the age ranges were the following: (i) ORG samples for LC-MS and WB analyses of proteins, LC-MS and TLC analyses of lipids (subjects ranging from 61 to 93 years of age); (ii) TIS-NM samples for LC-MS analyses of proteins, LC-MS and TLC analyses of lipids (isolated from pooled SN of subjects ranging from 48 to 92 years of age); (iii) ORG-NM samples for LC-MS analyses of proteins (subjects ranging from 60 to 82 years of age); (iv) SN tissue lysates for WB analyses of proteins (subjects ranging from 48 to 89 years of age); and (v) SN tissue slices for IEM (subjects ranging from 63 to 91 years of age). For each type of preparation intended for a specific determination, we have used tissues with similar (as much as possible) *post mortem* intervals to use the most reproducible conditions. It is noteworthy that all the analyses described in this study were performed on sets of samples coming from subjects with overlapping age ranges.

Indeed, we were studying brain aging and wanted to investigate elderly subjects, and in this age range the amount of NM accumulated in SN was sufficient for our determinations. Furthermore, we employed samples from subjects in this age range since it was difficult to collect brain samples in a narrower age range.

### Isolation of NM-containing organelles, NM purified from SN tissues, and NM purified from NM-containing organelles

The isolation of intact ORG samples from SN tissue of single subjects, or rarely from SN of two subjects depending on the availability of brain tissues, was performed immediately after SN dissection, in order to avoid freezing and thawing steps of tissues that otherwise would have altered the integrity of ORG samples. The isolation procedure of ORG samples was slightly modified and adapted for LC-MS analyses from our previously published protocol.^3^

TIS-NM samples were isolated from pooled SN tissues and prepared as previously reported.^3,9,42^

ORG-NM samples, consisting of the NM pigment fraction isolated from NM-containing organelles, were prepared from ORG samples and described as follows. Intact ORG samples suspended in their isolation buffer^3^ were processed with three freeze/thaw cycles (−78 °C/+37 °C) in order to disrupt organelles membranes. The sample was then centrifuged (17,500 × *g*, 15 min, 4 °C) to obtain a pellet containing the NM pigment. This pellet was subsequently washed twice in the same buffer, centrifuged as described above, and then treated for LC-MS analyses of proteins.

### Samples preparation for liquid chromatography-mass spectrometry analyses of proteins

Samples analyzed by LC-MS for proteomic analyses were isolated from the following subjects: (i) two ORG samples isolated from two different subjects (respectively 86 and 69 y.o.); (ii) two TIS-NM samples isolated from pooled SN tissues, the first one from a pool of five subjects (from 72 to 86 years of age) and the second one from pooled twelve subjects (from 70 to 92 years of age); and (iii) two ORG-NM samples, the first one isolated from one subject (82 y.o.) and the second one from two pooled subjects (60 and 69 y.o.).

TIS-NM samples (dried NM pigments) were hydrated in bi-distilled water under gentle shaking for 3 days in order to obtain a homogeneous suspension in water for tryptic digestion. The resulting TIS-NM suspension was then concentrated (Concentrator 5301; Eppendorf, Hamburg, Germany). ORG samples were concentrated in their isolation buffer while ORG-NM was directly treated with RapiGest SF (Waters, Milford, MA, USA) as detailed below.

In order to dissolve membrane proteins and break NM pigment in all samples, a solution of surfactant RapiGest in 100 mM (pH 7.9) ammonium bicarbonate was added to ORG, TIS-NM and ORG-NM samples according to the manufacturer’s protocol. A final concentration of 0.25 % (v/v) of RapiGest was adjusted with 100 mM ammonium bicarbonate (pH 7.9). Mixtures were then heated at 100 °C for 5 min, cooled to room temperature and digested overnight at 37 °C by adding sequencing grade modified trypsin (Promega, Madison, WI, USA) at an enzyme/substrate ratio of 1:50 (w/w). An additional aliquot of 0.5 µg trypsin was added in the morning and digestion was then prolonged for 4 h. The addition of 0.5 % trifluoroacetic acid stopped the enzymatic reaction and subsequent incubation at 37 °C for 45 min completed the acidic hydrolysis of RapiGest. The water insoluble degradation products were removed by centrifuging at 13,000 × *g* for 10 min and supernatants containing digested proteins were desalted using PepClean C-18 spin columns (Pierce Biotechnology, Inc., Rockford, IL, USA), concentrated and finally suspended in 20 µl of 0.1 % (v/v) formic acid.

### Liquid chromatography-mass spectrometry analyses of proteins

Three different types of samples (ORG, TIS-NM, ORG-NM) were prepared from SN tissues (Supplementary Fig. 1): two samples for ORG, two samples for TIS-NM, and two samples for ORG-NM as described in the previous paragraph. Each of the two ORG samples was injected three times for LC-MS analysis, each of the two TIS-NM samples was injected three times, while one ORG-NM sample was injected two times and the other one was injected only one time due to its scarce amount.

The multidimensional protein identification technology (MudPIT) system^46,136^ is a 2DC-MS/MS platform, composed of a two dimensional micro-high performance liquid chromatography system (Surveyor HPLC; Thermo Fisher Scientific, Inc., San Jose, CA, USA) coupled online to a mass spectrometer, using ProteomeX-2 configuration (Thermo Fisher Scientific, Inc.). Briefly, digested peptide mixtures were loaded onto a capillary strong cation exchange column (Biobasic-SCX column, 0.32 i.d. × 100 mm, 5 µm; Thermo Fisher Scientific, Inc.) and eluted stepwise with ammonium chloride injections of increasing molarity (5, 10, 15, 20, 30, 40, 80, 120, 400, 600, 700 mM). Fractions were captured in turn onto peptide traps (Zorbax 300 SB-C18, 0.3 i.d. × 5 mm, 5 µm, 300 Å; Agilent Technologies, Santa Clara, CA, USA) for concentration and desalting prior to final separation by reversed phase C18 column (Biobasic-18, 0.180 i.d. × 100 mm, 5 µm, 300 Å; Thermo Fisher Scientific, Inc.). Peptides were eluted using an acetonitrile gradient (eluent A, 0.1 % formic acid in water; eluent B, 0.1 % formic acid in acetonitrile): the gradient profile was 5 % eluent B for 3 min, 5–40 % B in 50 min, 40–80 % B in 10 min, 80–95 % B in 5 min, 95 % B in 10 min.

The flow rate was 100 μl/min, which was split to achieve a final flux of 2 μl/min. Then, eluting peptides were electrosprayed directly into a hybrid ion trap-Orbitrap mass spectrometer (LTQ Orbitrap XL^TM^ ETD; Thermo Fisher Scientific, Inc.), equipped with a nanospray ion source. The spray capillary voltage was set at 1.5 kV, and the ion transfer capillary temperature was maintained at 220 °C. For each step of peptide elution from C18 column, full mass spectra were recorded in the positive ion mode over a 400–1600 m/z range, with a resolving power of 60,000 (full width at half-maximum) and a scan rate of 2 spectra/s. This step was followed by four low-resolution MS/MS events that were sequentially generated in a data-dependent manner on the top four ions selected from the full MS spectrum, using dynamic exclusion for the MS/MS analysis. In particular, the MS/MS scans were acquired by setting a normalized collision energy of 35 % on the precursor ion and, when a peptide ion was analyzed twice, applying an exclusion duration of 0.5 min. Mass spectrometer scan functions and high performance liquid chromatography solvent gradients were controlled by the Xcalibur data system version 1.4 (Thermo Fisher Scientific, Inc.).

### Identification of proteins detected by liquid chromatography-mass spectrometry analyses

Protein identification was carried out by matching experimental spectra to peptide sequences using the SEQUEST database search algorithm, contained in BioWorks version 3.3.1 SP1 (University of Washington, licensed to Thermo Fisher Scientific, Inc.) using SEQUEST PC Cluster.^137^ For peptide matching, an updated non-redundant human protein sequence database of 276,790 entries, downloaded in January 2009 from the National Center for Biotechnology Information (NCBI) website (http://www.ncbi.nlm.nih.gov), was used. In addition, to make a thorough comparison of our data with data obtained from other studies^23,24,38^ and to avoid possible bias and false results due to the use of not-aligned protein codes, the GI accession numbers of identified proteins were updated to those downloaded in April 2017 from the UniProt repository (http://www.uniprot.org/). Therefore, for these comparisons, we have considered only one time the few proteins we identified in our samples by two different GI accession numbers but with same UniProt accession number.

Known abundant contaminating proteins such as keratins, trypsin and typical abundant proteins of red blood cells (rarely observed as probable contaminants in ORG samples) were removed prior to the final data analysis, referring to published proteomic data sets of red blood cells.^47,48^ HLA peptides were identified using the updated non-redundant human HLA isoform (*HLA* gene) database^20^ downloaded from NCBI.

This analysis enabled the identification of peptide sequences and related proteins. Since the confidence of protein identification depends on the stringency of the identification of the peptide sequence and peptide matching, particularly when using data from a single peptide, a high stringency was guaranteed by using the following method. The peptide mass search tolerance was set to 1.00 Da; the precursor ion tolerance was set to 50 ppm and the intensity threshold was set to 100. Moreover, all searches were performed with no enzyme and in order to assign a final score to the proteins, the SEQUEST output data were filtered by setting the peptide probability to 1 × 10^−3^, the minimum correlation score values (Xcorr) was chosen greater than 1.5, 2.0, 2.5, and 3.0 for single-, double-, triple-, and quadruple-charged ions respectively, and the consensus score higher than 10. False-positive peptides ratio, calculated through reverse database, was less than 5 %. For decoy searches a reversed version of the target human protein database was generated using the reverse database function in Bioworks 3.3.1 software (Thermo Fisher Scientific, Inc.). The number of peptides, notably the SpC, detected by MS/MS was utilized as indicator of relative protein abundance. The group of representative proteins was selected considering proteins detected by SpC ≥ 2 as average value in at least one of the three types of samples.

The area-proportional Euler diagrams were calculated using the eulerAPE drawing tool (software at http://www.eulerdiagrams.org/eulerAPE/).^138^ Analyses of proteins for Euler diagram calculation in ORG, TIS-NM, and ORG-NM, as shown in Fig. 3, were performed by using NCBI accession (GI number). Euler diagram calculation in the comparison of our list of proteins with those previously reported for NM-containing organelles of human SN^23,24^ and for human brain lysosomes^38^ were performed by using UniProt accession number.

Individual cellular location was assigned to each protein according to the GOA database (http://geneontology.org/), the UniProt database (http://www.uniprot.org/) and authors’ PubMed search. It should be noted that some proteins may have multiple cellular location: in these cases the most typical and representative cellular location was manually assigned. We were unable to assign the cellular location for some proteins which were therefore classified as “unknown cell location” and additional proteins without a complete characterization at the moment of data analysis were classified as “uncharacterized proteins”.

The names of genes and proteins described in the main text and supplementary files were retrieved from updated UniProt and UniParc database (April 2017), and we adopt as the protein name symbol that of the approved gene symbol.

### Samples preparation for liquid chromatography-mass spectrometry analysis of lipids

Samples analyzed by LC-MS for identification of lipids were isolated from the following subjects: (i) two TIS-NM samples isolated from pooled SN tissues, the first one isolated from a pool of seven subjects (from 71 to 85 years of age) and the second one from pooled four subjects (from 82 to 85 years of age); (ii) three ORG samples, two of which isolated from two different subjects (respectively 81 and 89 y.o.) and the third one from two pooled subjects (74 and 89 y.o.).

The extraction of total lipids from TIS-NM samples was performed by using methanol and hexane as previously reported.^3,9,42^ The organic fractions (methanol and then hexane) derived from NM pigments were combined and dried under nitrogen flow.

The total lipids were extracted from ORG samples with methanol and hexane, as for TIS-NM samples. The ORG samples in few µl of isolation buffer were resuspended in about 0.5 ml of methanol and then centrifuged (1000 × *g*, 30 min, 20 °C). The supernatant was collected and the remaining pellet was resuspended in about 0.5 ml of hexane, and subsequently centrifuged as described above. Again, organic fractions (methanol and then hexane) extracted from ORG samples were finally combined and dried under nitrogen flow.

### Liquid chromatography-mass spectrometry analysis of lipids

For LC-MS analysis, the dried lipids extracted from TIS-NM and ORG samples were dissolved in 100 µl chloroform and diluted 1:1 (v/v) in eluent A, as described below. Typically, 5 µl of sample were injected on a C8 reversed phase column (BioBasic C8, 100 × 0.18 mm, 5 µm, 300 Å; Thermo Fisher Scientific, Inc.), using a micro liquid chromatography system (Surveyor HPLC; Thermo Fisher Scientific, Inc.) coupled to a mass spectrometer with Orbitrap^TM^ mass analyzer (Exactive Plus; Thermo Fisher Scientific, Inc.), equipped with a nanospray ion source. Liquid chromatography was operated at a flow rate of 100 μl/min, split in order to achieve a final flux of 2 μl/min, with a gradient as follows: 100 % eluent A was held isocratically for 10 min, then linearly increased to 100 % B over 30 min and held at 100 % B for 12 min. Eluent A consisted of methanol-acetonitrile-aqueous 1 mM ammonium acetate (60:20:20, v/v/v). Eluent B consisted of isopropanol-acetonitrile (90:10, v/v).

Nanospray was achieved using a coated fused silica emitter (360 µm o.d./50 µm i.d. 730 µm tip i.d.; New Objective, Inc., Woburn, MA, USA) held to 1.5 kV. The heated capillary was held at 220 °C. Full mass spectra were recorded in negative and positive ion mode over a 400–2000 m/z range. The resolution was set to 100,000 with 2 microscans/s, restricting the Orbitrap loading time to a maximum of 50 ms with a target value of 5E5 ions (ultimate mass accuracy mode).

Manual interpretation of mass spectra was performed to evaluate the major lipid species present in the analyzed lipid extracts.

### Lipids extraction and partitioning for thin-layer chromatography analyses of lipids

Samples analyzed by TLC for identification and semiquantitative assessment of lipids were isolated from the following subjects: (i) four TIS-NM samples isolated from pooled SN tissues, the first three samples isolated from a pool of five subjects each (respectively from 48 to 85 years of age, from 67 to 85 years of age, from 73 to 85 years of age) and the fourth sample from pooled four subjects (from 62 to 86 years of age); (ii) three ORG samples, each of which isolated from one subject (respectively 62, 61, and 77 y.o.).

Total lipids extracts for TLC analyses were prepared from TIS-NM samples as previously described by using methanol and hexane.^3,9,42^ Additionally, these TIS-NM lipids extracts dried under nitrogen stream were then resuspended in chloroform-methanol-water (2:1:0.1, v/v/v) and subjected to a two-phase Folch’s partitioning, resulting in the separation of an aqueous phase containing gangliosides and an organic phase containing all other lipids, including glycerophospholipids, neutral glycosphingolipids and sphingomyelin.

The ORG samples in few µl of isolation buffer were snap frozen and then lyophilized. Lipids were extracted with chloroform-methanol-water (2:1:0.1, v/v/v). Then, total lipid extracts were subjected to a two-phase Folch’s partitioning.

Following the two-phase partitioning, organic phases deriving from total lipids extracted from both TIS-NM and ORG samples were analyzed by TLC, loading equivalent amounts of lipids. Lipids were separated by mono-dimensional high performance TLC silica gel using chloroform-methanol-0.2 % aqueous calcium chloride (60:35:8, v/v/v) as a solvent system. After separation, lipids were detected by spraying the TLC plates with anisaldehyde, a reagent for the general detection of lipids. Identification of lipids after separation and chemical detection was assessed by co-migration with lipid standards (Avanti Polar Lipids, Inc., Alabaster, AL, USA; Sigma-Aldrich Co., St. Louis, MO, USA; some standard were synthesized in laboratories of the Department of Medical Biotechnology and Translational Medicine, University of Milan, Segrate, Italy).

Cholera toxin staining of lipid present in the aqueous phases was performed as described hereafter. Briefly, after chromatographic running using chloroform-methanol-0.2 % aqueous calcium chloride (50:42:11, v/v/v) as a solvent system, the TLC plate was well dried and fixed with a polyisobutylmethacrylate solution prepared dissolving 1.3 g of polyisobutylmethacrylate in 10 ml chloroform and diluting 8 ml of this solution with 42 ml of hexane. The TLC plate was immersed three times in this solution and then allowed to dry for 1 h. The dried TLC was soaked for 30 min in 0.1 M Tris-hydrochloride (pH 8.0), 0.14 M sodium chloride containing 1 % bovine serum albumin. The TLC was then incubated with *Clostridium perfringens* sialidase (0.12 U/ml in 0.05 M acetate buffer pH 5.4 and 4 mM calcium chloride) overnight at room temperature. Afterward, the TLC was incubated with biotin-conjugated cholera toxin subunit B (10 μg/ml; Sigma-Aldrich Co.) in phosphate-buffered saline containing 1 % bovine serum albumin for 1 h, and subsequently for 1 h with horseradish peroxidase-conjugated streptavidin (2 μg/ml; Sigma-Aldrich Co.) in the same solution. After several washings with phosphate-buffered saline, the TLC plate was developed for 5 min with o-Phenylenediamine dihydrochloride substrate (Sigma-Aldrich Co.), 1 tablet in 50 ml citrate-phosphate buffer (pH 5.0) and 20 μl hydrogen peroxide.

### Electron microscopy

Transmission electron microscopy for morphological evaluation was performed on SN tissue slices and isolated ORG samples prepared according to our previously published method.^3^

IEM experiments were carried out on SN tissue blocks which were fixed for 2 h at 4 °C in a mixture of 4 % paraformaldehyde and 0.25 % glutaraldehyde in cacodylate buffer (0.12 M, pH 7.4). Tissue samples were extensively washed with cacodylate buffer, dehydrated in a graded ethanol series and then embedded in LRW resin. Ultra thin sections (80 nm) were prepared using a ultramicrotome (Leica Ultracut; Leica Microsystems GmbH, Wien, Austria), collected over nickel grids and incubated for 90 min at room temperature with primary antibodies diluted in phosphate-buffered saline (pH 7.4). The concentrations of primary antibodies used for IEM experiments were the following: APOD (1:50), ATP5G1 (1:100), ATP6V1B2 (1:100), CTSD (1:100), DHDDS (1:200), FTH1 (1:250), FTL (1:300), GPNMB (1:100), HLA (1:200), LAMP1 (1:20), LAMP2 (1:20), MAP1LC3B (1:50 for antibody from Cell Signaling Technology; 1:25 for antibody from Abgent Inc.), PLBD2 (1:100), RAB5A (1:100), SCARB2 (1:50), SNCA (1:200), SQSTM1 (1:30), SRD5A3 (1:25), and UBA52 (1:250). The grids were then washed with phosphate-buffered saline and incubated for 60 min with gold-conjugated secondary antibodies (British Biocell International, Cardiff, UK) diluted 1:100 in phosphate-buffered saline. After extensive washing, the grids were post fixed with 1 % glutaraldehyde in cacodylate buffer. Samples were then stained with saturated uranyl acetate in water for 5 min, washed and then stained with 3 mM lead citrate for 5 min. Finally, the sections were photographed using a transmission electron microscope LEO 912AB (Carl Zeiss AG, Oberkochen, Germany) equipped with a Proscan CCD camera (ProScan, Lagerlechfeld, Germany) controlled by EsivisionPro software (Soft Imaging System, Münster, Germany). Electron microscopy was performed at the Advanced Light and Electron Microscopy BioImaging Center—San Raffaele Scientific Institute.

Each protein was investigated by IEM in at least one to three samples deriving from different subjects, depending on SN tissue availability. Data of subjects analyzed by IEM are reported in the legends of figures chosen as representative of all electron microscopy experiments. Each IEM experiment was routinely checked for negative controls as described below: (i) the reaction was performed on SN tissue slices without either primary or secondary antibody to confirm the absence of gold particles in the examined sections; (ii) in the presence of primary and secondary antibodies, the absence of gold particles was verified in the resin devoid of tissue; and (iii) the most relevant control we did was to check the absence of signal in cellular/tissue components where it was not expected. Only after passing these three negative controls, we evaluated the signal in NM-containing organelles which we judged as specific even if represented by few gold particles.

### Western blotting analyses of tissue lysates and NM-containing organelles

SN tissues were homogenized and sonicated with five volumes of lysis buffer as described above. In case of soluble proteins, the lysis buffer was composed of 50 mM Tris-hydrochloride (pH 7.5), 150 mM sodium chloride, 0.1 % sodium dodecyl sulfate, 1 % Triton® X-100, and 1 % protease inhibitor cocktail (Sigma-Aldrich Co.). For membrane-bound proteins, the lysis buffer was composed of phosphate-buffered saline (pH 7.4), 0.1 % sodium dodecyl sulfate, 1 % Triton® X-100, and 1 % protease inhibitor cocktail. After short sonication cycles in ice, SN homogenates were centrifuged at 17,500 × *g*, 4 °C (30 min for soluble proteins, 10 min for membrane-bound proteins) and the supernatants were collected. From supernatants, proteins were precipitated by acid treatment to remove components of buffers that could influence protein quantitation. Then, Lowry method was performed on the protein pellets to measure the total protein concentration in both SN tissue lysates and ORG samples.

For electrophoresis, samples were heated in reducing sample buffer and then loaded onto 0.75 mm thick polyacrylamide mini gels (different pore sizes, depending on the desired separation of proteins) and separated by one-dimensional sodium dodecyl sulfate-polyacrylamide gel electrophoresis (Mini-PROTEAN® 3 Cell; Bio-Rad Laboratories, Inc., Hercules, CA, USA). Prestained protein markers (New England BioLabs, Inc., Ipswich, MA, USA) were used as size standards in the electrophoresis. Proteins were then over-night transferred (Mini Trans-Blot® Electrophoretic Transfer Cell; Bio-Rad Laboratories, Inc.) to 0.45 µm supported nitrocellulose membranes (Hybond®-C Extra; Amersham Biosciences, Little Chalfont, UK). Ponceau-S staining of membranes was performed to check the efficiency of proteins transfer.

For immunoblotting, membranes were blocked for 2 h at room temperature with fatty acid/globulin-free bovine serum albumin (Sigma-Aldrich Co.) or skimmed milk, with concentrations ranging from 3 to 8 %, depending on the properties of investigated protein, antibody specificity, etc., in phosphate-buffered saline (pH 7.4) with 0.1 % Tween® 20. Then membranes were incubated with primary antibodies diluted in blocking solutions, while temperature and time of incubation varied according to antibodies specificity. The concentrations of primary antibodies used for WB experiments were the following: APOD (1:300), ATP5G1 (1:100), ATP6V1B2 (1:400), CTSD (1:500), DHDDS (1:1000), FTH1 (1:3500), FTL (1:2000), GPNMB (1:1500), LAMP1 (1:50), LAMP2 (1:200), MAP1LC3B (1:600), PLBD2 (1:300), RAB5A (1:1000), SCARB2 (1:600), SNCA (1:1500), SQSTM1 (1:300), SRD5A3 (1:100), and UBA52 (1:200). After washing, membranes were incubated for 90 min at room temperature with horseradish peroxidase-labeled secondary antibodies with a concentration range 1:2000–1:5000 in block solution (Jackson ImmunoResearch, Inc., West Grove, PA, USA; EMD Millipore). After extensive washing, the immunoreactive species were visualized by SuperSignal® West Pico chemiluminescent substrate as described in the manufacturer’s protocol (Pierce Biotechnology, Inc.). The films were exposed for few seconds up to 15 min. As a control for aspecific binding, the same procedure was executed without primary antibody. When multiple blotting of membranes was necessary, as in case of loading control with CTSD antibody, the Restore^TM^ PLUS Western Blot Stripping Buffer (Pierce Biotechnology, Inc.) was used according to manufacturer’s protocol, and successful stripping was confirmed by re-incubating the membranes with the chemiluminescent substrate.

The WB analyses in this study are qualitative rather than quantitative. For the electrophoresis separation of proteins in the samples, we kept an empty lane between lanes containing SN tissue lysates and those containing ORG samples in order to avoid contamination leading to false positive results. For the preparation of figures, we divided the two lanes of interest by a thin white line between juxtaposed lanes. It should be noted that: (i) ORG samples were loaded at high volumes, due to their low total protein concentration with respect to SN tissue lysates, and because the concentration procedure of ORG sample produced an insoluble and untreatable residue from which proteins could be no longer recovered (high salt composition of the isolation buffer, particular nature of the NM pigment contained in ORG samples, etc.); (ii) the granular composition of the NM pigment present in ORG samples could interfere with the initial electrophoresis run in the stacking gel, thus leading to lateral diffusion of proteins; and (iii) the contamination of proteins from SN tissue lysate (largely rich of proteins) to ORG sample was less probable but could not be excluded and lead as well to false positive results in ORG samples. The total amounts of proteins loaded into each lane of the gel were then chosen depending on the availability of the sample (particularly ORG sample), and on the content of each selected protein in both SN tissue lysates and ORG samples. The ratios of protein content between SN tissue lysates and ORG samples for each protein analyzed by WB are reported in the figure legends. Note that for ATP6V1B2, GPNMB, and RAB5A proteins, the WB analyses were performed on SN tissue lysates and ORG samples on different days, due to the lack of either SN tissues or ORG samples, depending on human brain tissues availability, and it was sometimes impossible to run all types of samples at the same time. Relative lanes where then juxtaposed as previously explained. The use of WB on SN tissue lysates was chiefly employed to assess the specificity of each antibody for the target protein as essential information for IEM experiments, and to verify its presence or absence in the ORG samples (*n* ≥ 3 for each protein). Data of subjects from which SN tissue lysates and ORG samples were prepared are reported in the legends of figures chosen as representative of all WB experiments performed on different samples.

### Data availability

All relevant data are within the paper and its Supporting Information files. However, raw data supporting the results reported in this article are available upon request.

## Electronic supplementary material

Supplementary Data 1

Supplementary Data 2

Supplementary Data 3

Supplementary Data Legends

Supplementary Tables

Supplementary Figures
