# Supplementary material for: Neuromelanin organelles are specialized autolysosomes that accumulate undegraded proteins and lipids in aging human brain and are likely involved in Parkinson’s disease
Source: NPJ Parkinsons Dis. 2018 Jun 5;4:17. doi: 10.1038/s41531-018-0050-8 (PMC5988730; doi:10.1038/s41531-018-0050-8)
Supplement: Supplementary file 4 — Supplementary Data Legends [file 41531_2018_50_MOESM4_ESM.docx]

**Neuromelanin organelles are specialized autolysosomes that accumulate undegraded proteins and lipids in aging human brain and are likely involved in Parkinson's disease**

Fabio A. Zucca^1*^, Renzo Vanna^1,2*^, Francesca A. Cupaioli^1^, Chiara Bellei^1^, Antonella De Palma^1^, Dario Di Silvestre^1^, Pierluigi Mauri^1^, Sara Grassi^3^, Alessandro Prinetti^3^, Luigi Casella^4^, David Sulzer^5,6,7^, Luigi Zecca^1,5#^

* These authors contributed equally to this work.

^1^ Institute of Biomedical Technologies, National Research Council of Italy, Segrate (Milan), Italy; ^2^ IRCCS Don Carlo Gnocchi ONLUS Foundation, Milan, Italy; ^3^ Department of Medical Biotechnology and Translational Medicine, University of Milan, Segrate (Milan), Italy; ^4^ Department of Chemistry, University of Pavia, Pavia, Italy; ^5^ Department of Psychiatry, Columbia University Medical Center, New York State Psychiatric Institute, New York, NY, USA; ^6^ Department of Neurology, Columbia University Medical Center, New York, NY, USA; ^7^ Department of Pharmacology, Columbia University Medical Center, New York, NY, USA.

# Corresponding author:

Dr. Luigi Zecca

Institute of Biomedical Technologies - National Research Council of Italy

Via Cervi, 93 - 20090 Segrate (MI), Italy

Tel. +39 02 26422616; Fax +39 02 26422660

Email: [luigi.zecca@itb.cnr.it](mailto:luigi.zecca@itb.cnr.it)

**This file includes:**

- **Legends of Supplementary Data 1-3;**
- **Supplementary References.**

**Supplementary Data 1.** List of all proteins detected (1020 in total) in ORG, TIS-NM, and ORG-NM samples. Each type of sample was prepared in duplicate and then analyzed by multiple LC-MS analyses. For details of subjects and preparation of samples for LC-MS analysis of proteins see Methods. For each protein, NCBI accession (GI number), UniProt accession number, protein name, gene name, cellular location, and details of LC-MS analyses are reported. A comparison of the list of proteins we detected with those previously reported by proteomic analyses on NM-containing organelles and human brain lysosomes is summarized in the following columns: (i) one column containing the proteins found also within the 72 proteins identified by Tribl and colleagues;^1^ two columns containing the proteins found also within 1000 proteins and within the 166 significantly overrepresented proteins, both reported by Plum and colleagues;^2^ the column containing proteins found also within 48 human soluble lysosomal proteins plus 43 novel and potential lysosomal proteins (the latter group indicated by "a" in superscript).^3^ For each protein the most typical and representative cellular location was assigned. The first 293 lines (marked with X in the "Representative proteins" column) correspond to representative proteins detected by SpC ≥ 2 as average value in at least one of the three types of samples. Full colored cells correspond to representative proteins, light colored cells are related to non-representative proteins. The "Average score" is the average of all score values obtained from each LC-MS analysis, while the "Average # SpC" is the average value of total spectra identified for each protein. The column "Overall results" refers to overall proteins present in all three types of samples that are considered as a single dataset; the "Overall frequency" represents the number of sample types in which a specific protein was detected.

**Supplementary Data 2.** List of all HLA proteins detected in the three types of samples (ORG, TIS-NM and ORG-NM). These data were obtained by matching experimental spectra to peptide sequences of an updated non-redundant human database specific for *HLA* isoform (*HLA* gene) downloaded from NCBI ([http://www.ncbi.nlm.nih.gov](http://www.ncbi.nlm.nih.gov/)). Detailed analyses of HLA-related genes and alleles have been recently reported.^4^ The "Average score" is the average of all score values obtained from each LC-MS analysis, while the "Average # SpC" is the average value of total spectra identified for each protein. The column "Overall results" refers to overall HLA proteins present in all three types of samples which are considered as a single dataset; the "Overall frequency" represents the number of sample types in which a specific HLA protein was detected.

**Supplementary Data 3.** List of proteins detected by LC-MS in human brain lysosomes by purifying only the soluble resident lysosomal proteins using mannose 6-phosphate (Man-6-P) as an univocal lysosomal marker. The first 48 entries represent the known lysosomal proteins identified in lysosomes purified from human brain,^3^ with UniProt accession number, protein name and gene names up to date as of April 2017 from the UniProt repository, together with main E.C. classifications (data from Table 1 in Sleat and colleagues).^3^ Additionally, 43 proteins that were suggested as candidates of novel lysosomal proteins are indicated in the lower part of the list, updated in detail as previously described (data from Table 2 in Sleat and colleagues).^3^ The right part of the Data S3 table shows the proteins (with LC-MS details extrapolated from Supplementary Data 1) we detected in each type of sample (ORG, TIS-NM, and ORG-NM), which were also identified by Sleat and colleagues.^3^

**Supplementary References**

1. Tribl, F. et al. "Subcellular proteomics" of neuromelanin granules isolated from the human brain. *Mol. Cell. Proteomics.* **4**, 945-957 (2005).
2. Plum, S. et al. Proteomic characterization of neuromelanin granules isolated from human substantia nigra by laser-microdissection. *Sci. Rep.* **6**, 37139 (2016).
3. Sleat, D. E., Zheng, H., Qian, M., & Lobel, P. Identification of sites of mannose 6-phosphorylation on lysosomal proteins. *Mol. Cell. Proteomics* **5**, 686-701 (2006).
4. Cebrián, C. et al. MHC-I expression renders catecholaminergic neurons susceptible to T-cell-mediated degeneration. *Nat. Commun.* **5**, 3633 (2014).
