# Supplementary material for: Neuromelanin organelles are specialized autolysosomes that accumulate undegraded proteins and lipids in aging human brain and are likely involved in Parkinson’s disease
Source: NPJ Parkinsons Dis. 2018 Jun 5;4:17. doi: 10.1038/s41531-018-0050-8 (PMC5988730; doi:10.1038/s41531-018-0050-8)
Supplement: Supplementary file 5 — Supplementary Tables [file 41531_2018_50_MOESM5_ESM.docx]

**Neuromelanin organelles are specialized autolysosomes that accumulate undegraded proteins and lipids in aging human brain and are likely involved in Parkinson's disease**

Fabio A. Zucca^1*^, Renzo Vanna^1,2*^, Francesca A. Cupaioli^1^, Chiara Bellei^1^, Antonella De Palma^1^, Dario Di Silvestre^1^, Pierluigi Mauri^1^, Sara Grassi^3^, Alessandro Prinetti^3^, Luigi Casella^4^, David Sulzer^5,6,7^, Luigi Zecca^1,5#^

* These authors contributed equally to this work.

^1^ Institute of Biomedical Technologies, National Research Council of Italy, Segrate (Milan), Italy; ^2^ IRCCS Don Carlo Gnocchi ONLUS Foundation, Milan, Italy; ^3^ Department of Medical Biotechnology and Translational Medicine, University of Milan, Segrate (Milan), Italy; ^4^ Department of Chemistry, University of Pavia, Pavia, Italy; ^5^ Department of Psychiatry, Columbia University Medical Center, New York State Psychiatric Institute, New York, NY, USA; ^6^ Department of Neurology, Columbia University Medical Center, New York, NY, USA; ^7^ Department of Pharmacology, Columbia University Medical Center, New York, NY, USA.

# Corresponding author:

Dr. Luigi Zecca

Institute of Biomedical Technologies - National Research Council of Italy

Via Cervi, 93 - 20090 Segrate (MI), Italy

Tel. +39 02 26422616; Fax +39 02 26422660

Email: [luigi.zecca@itb.cnr.it](mailto:luigi.zecca@itb.cnr.it)

**This file includes:**

- **Supplementary Tables 1 and 2 with legends.**

**Supplementary Table 1.** Cellular location of 293 representative proteins

| **Cellular location** | **All samples** | | | **ORG** | | | **TIS-NM** | | | **ORG-NM** | | |
| --- | --- | --- | --- | --- | --- | --- | --- | --- | --- | --- | --- | --- |
|  | **# prot.** | **Total # SpC** | **Rel. # SpC** | **# prot.** | **Total # SpC** | **Rel. # SpC** | **# prot.** | **Total # SpC** | **Rel. # SpC** | **# prot.** | **Total # SpC** | **Rel. # SpC** |
| Lysosome | 34 | 7916 | 60.17 % | 25 | 1624 | 42.83 % | 26 | 5447 | 73.22 % | 23 | 845 | 43.87 % |
| Membrane | 23 | 2019 | 15.35 % | 14 | 1056 | 27.85 % | 7 | 560 | 7.53 % | 9 | 403 | 20.92 % |
| Cytoplasm | 51 | 812 | 6.17 % | 23 | 239 | 6.30 % | 28 | 327 | 4.40 % | 21 | 246 | 12.77 % |
| Cytoskeleton | 28 | 675 | 5.13 % | 16 | 292 | 7.70 % | 14 | 222 | 2.98 % | 19 | 161 | 8.36 % |
| Endoplasmic reticulum | 9 | 307 | 2.33 % | 5 | 73 | 1.93 % | 6 | 164 | 2.20 % | 2 | 70 | 3.63 % |
| Synapse | 5 | 275 | 2.09 % | 1 | 15 | 0.40 % | 5 | 249 | 3.35 % | 2 | 11 | 0.57 % |
| Cytosol | 10 | 235 | 1.79 % | 6 | 33 | 0.87 % | 3 | 183 | 2.46 % | 5 | 19 | 0.99 % |
| Extracellular | 20 | 233 | 1.77 % | 12 | 84 | 2.22 % | 9 | 102 | 1.37 % | 6 | 47 | 2.44 % |
| Nucleus | 38 | 228 | 1.73 % | 22 | 95 | 2.51 % | 13 | 90 | 1.21 % | 16 | 43 | 2.23 % |
| Mitochondrion | 11 | 83 | 0.63 % | 5 | 35 | 0.92 % | 6 | 46 | 0.62 % | 1 | 2 | 0.10 % |
| Uncharacterized protein | 17 | 75 | 0.57 % | 13 | 57 | 1.50 % | 4 | 10 | 0.13 % | 3 | 8 | 0.42 % |
| Vesicles | 7 | 70 | 0.53 % | 5 | 58 | 1.53 % | 2 | 7 | 0.09 % | 1 | 5 | 0.26 % |
| Immunoglobulin | 9 | 54 | 0.41 % | 6 | 42 | 1.11 % | 2 | 7 | 0.09 % | 3 | 5 | 0.26 % |
| Cell membrane | 12 | 49 | 0.37 % | 7 | 22 | 0.58 % | 3 | 18 | 0.24 % | 3 | 9 | 0.47 % |
| Melanosome | 1 | 49 | 0.37 % | 1 | 18 | 0.47 % | 1 | 3 | 0.04 % | 1 | 28 | 1.45 % |
| Unknown cell. location | 8 | 34 | 0.26 % | 7 | 22 | 0.58 % | 0 | 0 | 0 % | 4 | 12 | 0.62 % |
| Ribosome | 7 | 32 | 0.24 % | 5 | 20 | 0.53 % | 1 | 4 | 0.05 % | 4 | 8 | 0.42 % |
| Golgi apparatus | 3 | 11 | 0.08 % | 1 | 7 | 0.18 % | 0 | 0 | 0 % | 2 | 4 | 0.21 % |
| **Total** | **293** | **13157** | **100 %** | **174** | **3792** | **100 %** | **130** | **7439** | **100 %** | **125** | **1926** | **100 %** |

**Supplementary Table 1.** Detailed comparison of cellular locations of the 293 representative proteins (described in Supplementary Data 1) detected by LC-MS in all analyzed samples (ORG, TIS-NM and ORG-NM), as also represented in the histogram of Fig. 2. For details of subjects and preparation of samples for LC-MS analysis of proteins see Methods. The samples here represented correspond to those shown in Fig. 2 and here cellular compartments are ordered following the "Rel. # SpC" in "All samples". The "All Samples" column refers to overall representative proteins commonly present in all three types of samples which are considered as a single dataset. Note that some proteins have multiple cellular locations: for each protein the most typical and representative cellular location was assigned. The term "Vesicles" refers to vesicle trafficking (transport, fusion, etc.); the category "Unknown cell. location" consists of proteins for which a cellular location was still unclear, while the class "Uncharacterized proteins" comprises proteins for which a complete characterization was still missing. The "# prot." is the number of proteins belonging to a specific cellular compartment identified in each sample. The "Total # SpC" is the total number of SpC detected for each class of proteins. The "Rel. # SpC", in %, is the total number of SpC for a specific class of proteins (e.g., lysosomal) referred to the overall number of SpC of representative proteins of each sample, and this value represents the relative abundance of a particular class of proteins. The "Rel. # SpC" values have been approximated to the second decimal place.

**Supplementary Table 2**. LC-MS results of molecular species detected in lipid extracts from TIS-NM and ORG samples in negative and positive ion mode

| **Lipid molecules** | **Proposed structure** | **Ions observed (m/z)** | **TIS-NM** | **ORG** |
| --- | --- | --- | --- | --- |
| GD1 | (d18:1/18:0) | 917.5 [M - 2H]^2-^ | X | X |
|  | (d20:1/18:0) | 931.5 [M - 2H]^2-^ | X | X |
| GT1b | (d18:1/18:0) | 1077.5 [M - 2H]^2-^ | X |  |
|  | (d18:1/18:0) | 718 [M - 3H]^3-^ | X |  |
|  | (d20:1/18:0) | 708.6 [M - 3H]^3-^ | X |  |
| GM1 | (d18:1/18:0) | 773.5 [M - 2H]^2-^ | X |  |
| GD3 | (d18:1/18:0) | 734.9 [M - 2H]^2-^ | X |  |
|  | (d18:1/20:0) | 748.9 [M - 2H]^2-^ | X |  |
| LacCer | (t18:0/18:0) | 906.6 [M - H]^-^ | X |  |
| PS | (36:1) | 788.5 [M - H]^-^ | X | X |
|  | (38:6) | 806.5 [M - H]^-^ | X |  |
|  | (38:1) | 816.6 [M - H]^-^ | X |  |
|  | (40:6) | 834.5 [M - H]^-^ | X | X |
| PC | (36:1) | 786.5 [M - H]^-^ |  | X |
| PI | (38:4) | 885.5 [M - H]^-^ | X | X |
| ST | (t18:1/22:0) | 878.6 [M - H]^-^ | X |  |
|  | (d18:1/24:1) | 888.6 [M - H]^-^ | X |  |
|  | (d18:0/24:0) | 892.6 [M - H]^-^ | X |  |
|  | (d18:1/25:1) | 902.6 [M - H]^-^ | X |  |
|  | (d18:1/25:0) | 904.6 [M - H]^-^ | X | X |
|  | (d18:0/26:0) | 918.6 [M - H]^-^ | X |  |
|  | (d18:1/26:1) | 932.6 [M - H]^-^ | X |  |
| SM | (d18:1/16:0) | 703.5 [M + H]^+^ | X |  |
|  | (d18:1/16:0) | 725.5 [M + Na]^+^ | X |  |
|  | (d18:1/18:0) | 731.6 [M + H]^+^ | X |  |
|  | (d18:1/18:0) | 753.5 [M + Na]^+^ | X |  |
|  | (d18:1/20:0) | 759.6 [M + H]^+^ | X |  |
|  | (d18:1/20:0) | 781.5 [M + Na]^+^ | X |  |
|  | (d18:1/22:0) | 809.6 [M + Na]^+^ | X |  |
|  | (d18:1/24:0) | 815.5 [M + H]^+^ | X |  |
|  | (d18:1/24:0) | 837.6 [M + Na]^+^ | X |  |
| FA | (14:1) | 225.2 [M - H]^-^ | X |  |
|  | (14:0) | 227.2 [M - H]^-^ | X |  |
|  | (16:1) | 253.2 [M - H]^-^ | X |  |
|  | (16:0) | 255.2 [M - H]^-^ | X |  |
|  | (18:1) | 281.2 [M - H]^-^ | X |  |
|  | (18:0) | 283.2 [M - H]^-^ | X |  |

**Supplementary Table 2.** Summary of the main lipid species, in addition to dolichols and dolichoic acids (Fig. 6), detected by high resolution LC-MS analyses of lipid extracts from TIS-NM and ORG samples. For details of subjects and preparation of samples for LC-MS analysis of lipids see Methods. In particular, we reported for each of the m/z ions observed in the full MS spectrum (i) the proposed structure, (ii) the corresponding molecular species and (iii) in which type of sample they were identified. Moreover, regarding ganglioside GD1 it was not possible to assign the isoform "a" or "b", like in TLC analysis, because this attribution can be done only by observing specific fragment ions that were not recorded in these analyses. Abbreviations used in this table: FA = free fatty acids; GD1, GM1, GT1b, GD3 = gangliosides GD1, GM1, GT1b, GD3; LacCer = lactosylceramide; PC = phosphatidylcholine; PI = phosphatidylinositol; PS = phosphatidylserine; SM = sphingomyelin; ST = sulfatides.
