# Supplementary material for: Neuromelanin organelles are specialized autolysosomes that accumulate undegraded proteins and lipids in aging human brain and are likely involved in Parkinson’s disease
Source: NPJ Parkinsons Dis. 2018 Jun 5;4:17. doi: 10.1038/s41531-018-0050-8 (PMC5988730; doi:10.1038/s41531-018-0050-8)
Supplement: Supplementary file 6 — Supplementary Figures [file 41531_2018_50_MOESM6_ESM.docx]

**Neuromelanin organelles are specialized autolysosomes that accumulate undegraded proteins and lipids in aging human brain and are likely involved in Parkinson's disease**

Fabio A. Zucca^1*^, Renzo Vanna^1,2*^, Francesca A. Cupaioli^1^, Chiara Bellei^1^, Antonella De Palma^1^, Dario Di Silvestre^1^, Pierluigi Mauri^1^, Sara Grassi^3^, Alessandro Prinetti^3^, Luigi Casella^4^, David Sulzer^5,6,7^, Luigi Zecca^1,5#^

* These authors contributed equally to this work.

^1^ Institute of Biomedical Technologies, National Research Council of Italy, Segrate (Milan), Italy; ^2^ IRCCS Don Carlo Gnocchi ONLUS Foundation, Milan, Italy; ^3^ Department of Medical Biotechnology and Translational Medicine, University of Milan, Segrate (Milan), Italy; ^4^ Department of Chemistry, University of Pavia, Pavia, Italy; ^5^ Department of Psychiatry, Columbia University Medical Center, New York State Psychiatric Institute, New York, NY, USA; ^6^ Department of Neurology, Columbia University Medical Center, New York, NY, USA; ^7^ Department of Pharmacology, Columbia University Medical Center, New York, NY, USA.

# Corresponding author:

Dr. Luigi Zecca

Institute of Biomedical Technologies - National Research Council of Italy

Via Cervi, 93 - 20090 Segrate (MI), Italy

Tel. +39 02 26422616; Fax +39 02 26422660

Email: [luigi.zecca@itb.cnr.it](mailto:luigi.zecca@itb.cnr.it)

**This file includes:**

- **Supplementary Figures 1-9 with legends;**
- **Supplementary References.**


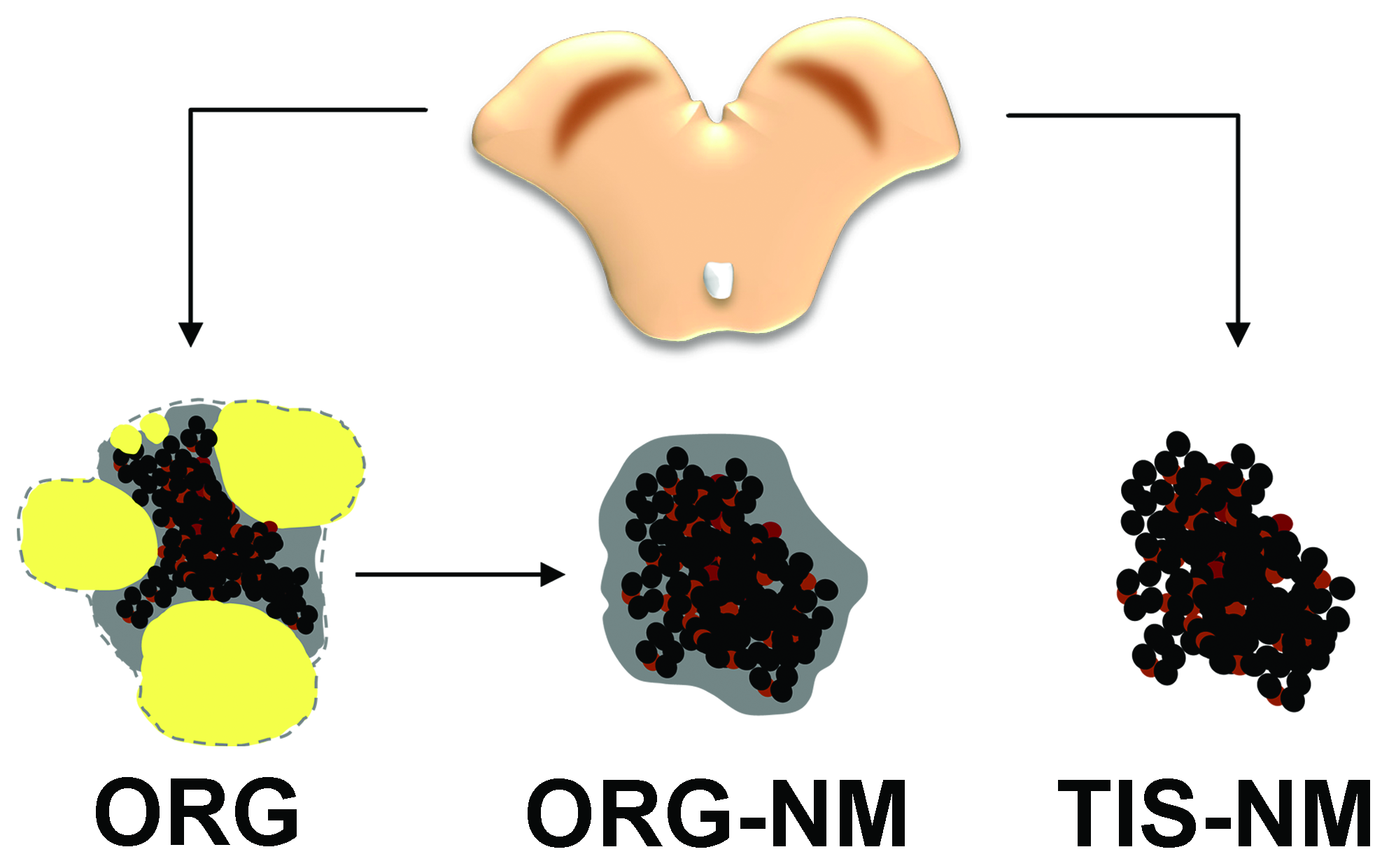


**Supplementary Fig. 1** Samples analyzed by LC-MS for identification of proteins. The following types of samples were prepared in duplicate from human SN: NM-containing organelles isolated from SN tissues immediately after dissection (ORG), NM pigment isolated from the above NM-containing organelles (ORG-NM), NM pigment isolated from pooled SN tissues (TIS-NM). For details of subjects and preparation of samples for LC-MS analysis of proteins see Methods.





**Supplementary Fig. 2** IEM of SN from healthy aged subjects for selected proteins. For number of IEM experiments see Methods. ATP5G1 (86 y.o.; gold particles = 20 nm). ATP6V1B2 (86 y.o.; gold particles = 20 nm). FTH1 (86 y.o.; gold particles = 15 nm). FTL (86 y.o.; gold particles = 20 nm). IEM for FTH1 and FTL were performed on the same SN tissue in order to have a direct comparison between the expression of these two isoforms. LAMP1 (86 y.o.; gold particles = 20 nm). PLBD2 (91 y.o.; gold particles = 15 nm). Lipid bodies are indicated by asterisks. NM pigment of the NM-containing organelles appears as black and electron dense granular aggregates. Scale bar in each panel = 250 nm.





**Supplementary Fig. 3** WB (for proteins detected by IEM in Supplementary Fig. 2) performed on SN tissue lysates and isolated ORG samples. For number of WB analyses see Methods. ATP5G1 (protein content ratio SN tissue lysate/ORG = 1.3). In SN tissue lysate (five pooled tissues, from 73 to 85 years of age) multiple bands were observed corresponding to aggregated forms, since the tendency of this protein to form oligomers has been noted:^1^ the two arrowheads indicate two aggregated forms (black arrowhead ~ 15 kDa; empty arrowhead ~ 24 kDa), while the bracket indicates aggregated forms at higher molecular weight (smear > 30 kDa) as previously reported.^1,2^ In ORG sample (isolated from one subject, 77 y.o.), only the higher molecular weight aggregates were present. We did not detect the very low molecular weight monomeric form of ATP5G1, presumably due to the low resolution of the electrophoresis apparatus.^1,3^ ATP6V1B2 (protein content ratio SN tissue lysate/ORG = 2.8). This protein was detected in SN tissue lysate (thirteen pooled tissues, from 62 to 86 years of age) and enriched in ORG sample (isolated from one subject, 81 y.o.), considering that the total protein content in ORG was 2.8-fold lower than that of SN tissue lysate. FTH1 (protein content ratio SN tissue lysate/ORG = 0.9). This protein was detected in SN tissue lysate only (eleven pooled tissues, from 54 to 85 years of age). Since FTH1 was not detected in ORG sample (isolated from one subject, 70 y.o.) by WB, a loading control was performed using CTSD antibody. FTL (protein content ratio SN tissue lysate/ORG = 0.9). The WB for FTH1 and FTL represented here were performed on the same ORG sample and SN tissue lysate to provide a direct comparison between the expression of the two isoforms. The WB analyses showed low levels of FTL in ORG, considering that the total protein content in ORG was quite similar to that of SN tissue lysate. LAMP1 (protein content ratio SN tissue lysate/ORG = 1.8). LAMP1 protein is highly enriched in SN tissue lysate (sixteen pooled tissues, from 48 to 85 years of age) if compared to ORG sample (isolated from one subject, 93 y.o.). PLBD2 (protein content ratio SN tissue lysate/ORG = 2.1). In the SN tissue lysate (five pooled tissues, from 73 to 85 years of age), the arrow indicates a band corresponding to the full mature protein (hp76, probably unglycosylated), and the two lower arrowheads indicate the doublet at lower molecular weight, as previously described by Jensen and colleagues,^4^ which correspond to differentially glycosylated forms of the C-term chain (the so-called 45 kDa C-term doublet). The other subunit formed by full protein maturation (small N-term chain) is not recognized by this antibody. In the ORG sample (isolated from one subject, 77 y.o.) only the differentially glycosylated forms of the 45 kDa C-term doublet seems to be present and highly enriched considering that the total protein content in ORG is 2.1-fold lower than that of SN tissue lysate.





**Supplementary Fig. 4** IEM of SN from healthy aged subjects for HLA and MAP1LC3B. For number of IEM experiments see Methods. HLA (89 y.o.; gold particles = 15 nm). WB was not performed for HLA since its presence has been thoroughly investigated.^5^ MAP1LC3B (63 y.o.; gold particles = 15 nm). This IEM was performed using a MAP1LC3B antibody different from that used for IEM and WB, as shown in Fig. 4 and Fig. 5 respectively, confirming the localization of this macroautophagic marker into NM-containing organelles. Lipid bodies are indicated by asterisks. NM pigment of the NM-containing organelles appears as black and electron dense granular aggregates. Scale bar in each panel = 250 nm.





**Supplementary Fig. 5** IEM of SN from healthy aged subjects for selected proteins (see Methods for details). APOD (91 y.o.; gold particles = 20 nm). DHDDS (63 y.o.; gold particles = 15 nm). In the higher magnification panel, signal for DHDDS was observed in the perinuclear space (nu = nucleus) of the endoplasmic reticulum (positive control), which was completely absent in NM-containing organelles. GPNMB (91 y.o.; gold particles = 15 nm). RAB5A (63 y.o.; gold particles = 15 nm). SQSTM1 (69 y.o.; gold particles = 20 nm). SRD5A3 (63 y.o.; gold particles = 15 nm). Positive control for SRD5A3 was found in the perinuclear space (nu = nucleus) of the endoplasmic reticulum, while signal was completely absent in NM-containing organelles. Lipid bodies are indicated by asterisks. NM pigment of the NM-containing organelles appears as black and electron dense granular aggregates. Scale bar in each panel = 250 nm.





**Supplementary Fig. 6** WB (for proteins detected by IEM of Supplementary Fig. 5) performed on SN tissue lysates and isolated ORG samples. For number of WB analyses see Methods. APOD (protein content ratio SN tissue lysate/ORG = 2.3). APOD protein is enriched in SN tissue lysate (nine pooled tissues, from 67 to 85 years of age) and in ORG sample (isolated from one subject, 66 y.o.). DHDDS (protein content ratio SN tissue lysate/ORG = 1.0). Since DHDDS was detected by WB in SN tissue lysate (thirteen pooled tissues, from 62 to 86 years of age) and not in ORG sample (isolated from one subject, 76 y.o.), a loading control was performed using CTSD antibody. GPNMB (protein content ratio SN tissue lysate/ORG = 2.6). In SN tissue lysate (thirteen pooled tissues, from 54 to 85 years of age) a typical pattern of bands was observed, probably showing the presence of different forms of GPNMB (mature, cleaved, uncleaved and variably glycosylated in a broad range between ~ 50 kDa and ~ 120 kDa or higher), although little is known about this recently characterized protein.^6^ The black arrowhead likely indicates the mature and highly glycosylated form, and the empty arrowhead likely indicates bands corresponding to the mature, N-term cleaved and non-glycosylated form (~ 50 kDa). The other subunit formed by GPNMB cleavage (short C-term) is not recognized by this antibody. In the ORG sample (isolated from one subject, 91 y.o.) an intense and medium-high molecular weight smear of GPNMB was observed (bracket), showing an enrichment of this protein in ORG where it is likely present in complex aggregates. RAB5A (protein content ratio SN tissue lysate/ORG = 2.6). The monomeric form of RAB5A was observed in SN tissue lysate (nine pooled tissues, from 67 to 85 years of age) and in ORG sample (isolated from one subject, 86 y.o.). SQSTM1 (protein content ratio SN tissue lysate/ORG = 2.2). SQSTM1 protein is enriched in SN tissue lysate (nine pooled tissues, from 67 to 85 years of age) and clearly present in ORG sample (isolated from one subject, 77 y.o.). SRD5A3 (protein content ratio SN tissue lysate/ORG = 1.0). As usual, since SRD5A3 was detected in SN tissue lysate (thirteen pooled tissues, from 62 to 86 years of age) and not in ORG sample (isolated from one subject, 86 y.o.), a loading control was performed using CTSD antibody.





**Supplementary Fig. 7** High performance TLC analysis of the aqueous phases obtained from lipid extracts. These lipids were extracted from TIS-NM sample (isolated from a pool of five subjects, from 73 to 85 years of age). The cholera toxin staining mainly revealed the presence of gangliosides GM1, GD1a, GD1b, and GT1b, which comprise the majority of total gangliosides in the adult brain. Abbreviations used in the figure: GD1a, GD1b, GD3, GM1, GM2, GM3, GT1b = gangliosides GD1a, GD1b, GD3, GM1, GM2, GM3, GT1b.


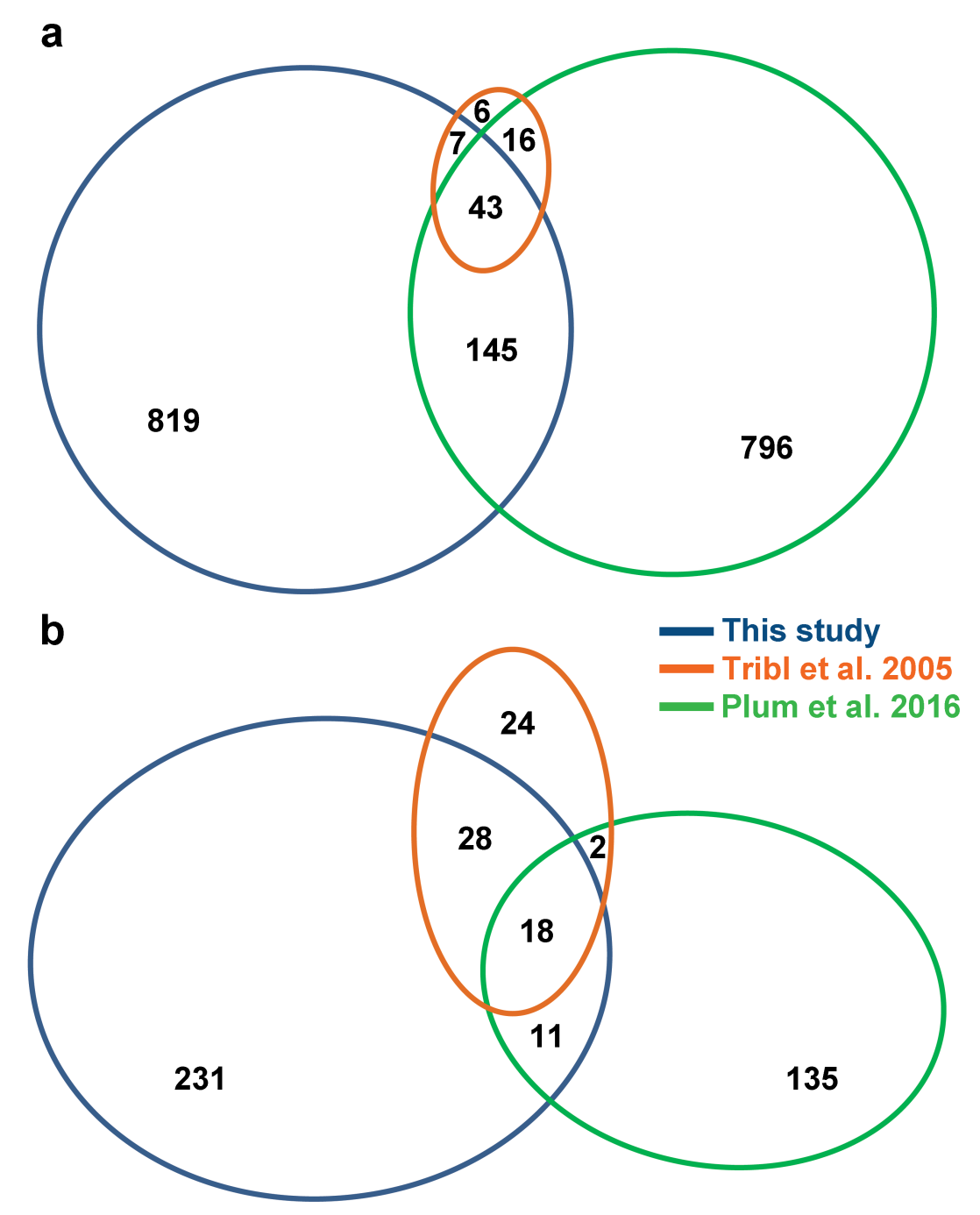


**Supplementary Fig. 8** Area-proportional Euler diagrams showing the proteins identified in this study compared with those reported by other proteomic analyses for NM-containing organelles. In panel **a** all proteins we identified in this study were compared with all proteins identified by Tribl and colleagues (72 proteins)^7^ and with all proteins identified by Plum and colleagues in at least 4/5 samples (1000 proteins).^8^ In panel **b** the comparison refers to the proteins detected as representative in this study: these were compared with all the proteins identified by Tribl and colleagues (72 proteins)^7^ and with proteins reported by Plum et al. as significantly overrepresented in NM-containing samples *vs.* control samples (166 proteins)^8^. These comparisons were made by matching the UniProt accession number of proteins identified in the three studies. Therefore, we have considered only one time the few proteins we identified in our samples by two different GI accession numbers but with same UniProt accession number.


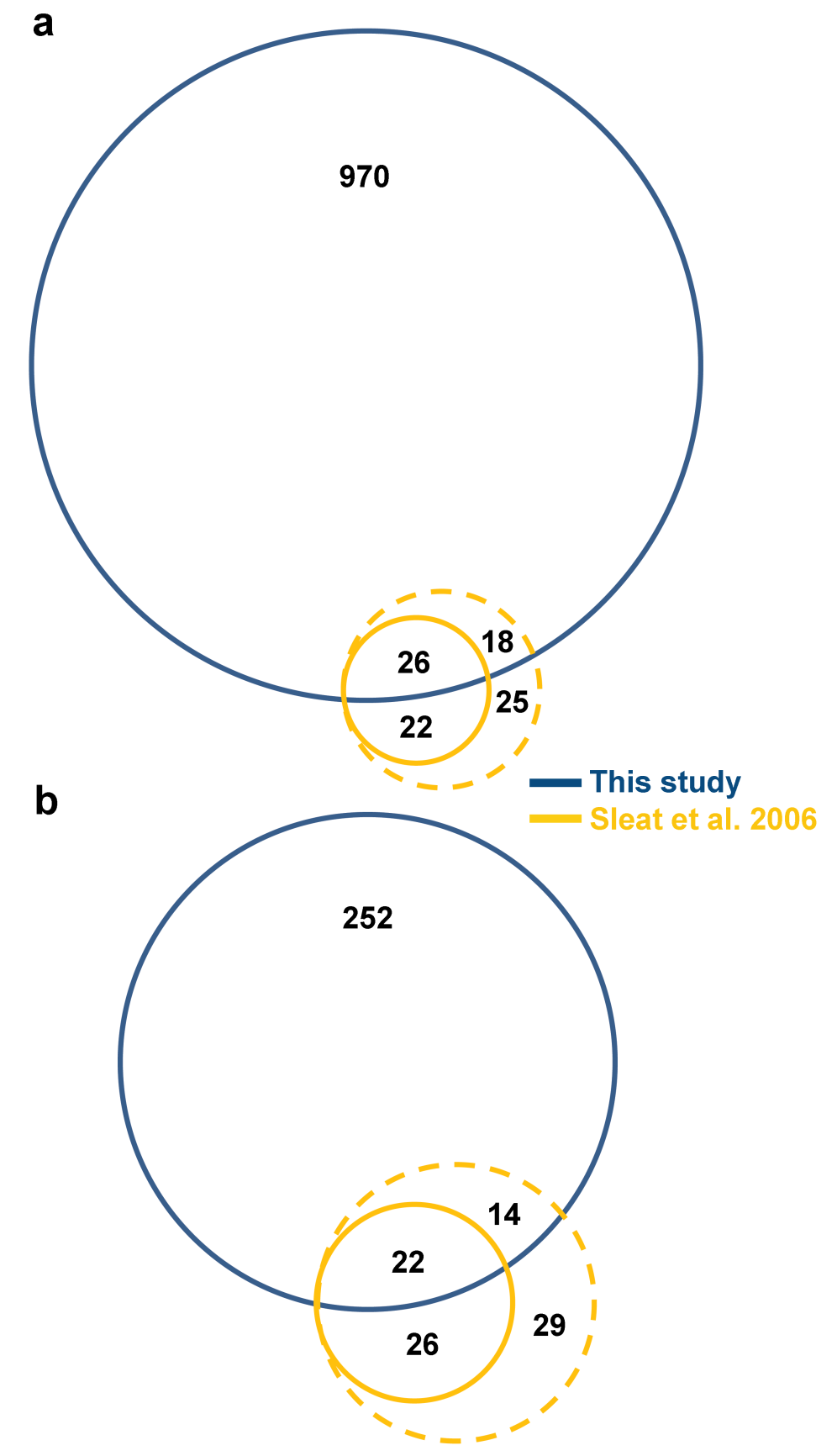


**Supplementary Fig. 9** Area-proportional Euler diagrams showing the proteins identified in this study compared with those identified in the soluble fraction of lysosomes purified from human brain (Man-6-P glycoproteome). In panel **a** the blue circle represents all proteins identified in this study, while in panel **b** it shows the representative proteins only. Sleat and colleagues reported soluble lysosomal proteins from human brain: among these proteins, 48 were confirmed as known lysosomal proteins (solid yellow circles), while 43 were classified as potentially novel lysosomal proteins (yellow dashed circles).^9^ Also in this case, the comparisons were made by matching the UniProt accession number of proteins identified in both studies. Again, we have considered only one time the few proteins we identified in our samples by two different GI accession numbers but with same UniProt accession number.

**Supplementary References**

1. Fearnley, I. M. et al. The sequence of the major protein stored in ovine ceroid lipofuscinosis is identical with that of the dicyclohexylcarbodiimide-reactive proteolipid of mitochondrial ATP synthase. *Biochem. J.* **268**, 751-758 (1990).
2. Hall, N. A., Lake, B. D., Dewji, N. N. & Patrick, A. D. Lysosomal storage of subunit c of mitochondrial ATP synthase in Batten's disease (ceroid-lipofuscinosis). *Biochem. J.* **275**, 269-272 (1991).
3. Kominami, E. et al. Specific storage of subunit c of mitochondrial ATP synthase in lysosomes of neuronal ceroid lipofuscinosis (Batten's disease). *J. Biochem.* **111**, 278-282 (1992).
4. Jensen, A. G. et al. Biochemical characterization and lysosomal localization of the mannose-6-phosphate protein p76 (hypothetical protein LOC196463). *Biochem. J.* **402**, 449-458 (2007).
5. Cebrián, C. et al. MHC-I expression renders catecholaminergic neurons susceptible to T-cell-mediated degeneration. *Nat. Commun.* **5**, 3633 (2014).
6. Hoashi, T. et al. Glycoprotein nonmetastatic melanoma protein b, a melanocytic cell marker, is a melanosome-specific and proteolytically released protein. *FASEB J.* **24**, 1616-1629 (2010).
7. Tribl, F. et al. "Subcellular proteomics" of neuromelanin granules isolated from the human brain. *Mol. Cell. Proteomics* **4**, 945-957 (2005).
8. Plum, S. et al. Proteomic characterization of neuromelanin granules isolated from human substantia nigra by laser-microdissection*. Sci. Rep.* **6**, 37139 (2016).
9. Sleat, D. E., Zheng, H., Qian, M. & Lobel, P. Identification of sites of mannose 6-phosphorylation on lysosomal proteins. *Mol. Cell. Proteomics* **5**, 686-701 (2006).
